# A Novel Active Learning Regression Framework for Balancing the Exploration-Exploitation Trade-Off

**DOI:** 10.3390/e21070651

**Published:** 2019-07-01

**Authors:** Dina Elreedy, Amir F. Atiya, Samir I. Shaheen

**Affiliations:** Computer Engineering Department, Cairo University, Giza 12613, Egypt

**Keywords:** active learning, exploration-exploitation, regression, optimization, mutual information, Kullback–Leibler divergence, entropy, query synthesis, demand learning, exploration-exploitation, sequential decision problems

## Abstract

Recently, active learning is considered a promising approach for data acquisition due to the significant cost of the data labeling process in many real world applications, such as natural language processing and image processing. Most active learning methods are merely designed to enhance the learning model accuracy. However, the model accuracy may not be the primary goal and there could be other domain-specific objectives to be optimized. In this work, we develop a novel active learning framework that aims to solve a general class of optimization problems. The proposed framework mainly targets the optimization problems exposed to the exploration-exploitation trade-off. The active learning framework is comprehensive, it includes exploration-based, exploitation-based and balancing strategies that seek to achieve the balance between exploration and exploitation. The paper mainly considers regression tasks, as they are under-researched in the active learning field compared to classification tasks. Furthermore, in this work, we investigate the different active querying approaches—pool-based and the query synthesis—and compare them. We apply the proposed framework to the problem of learning the price-demand function, an application that is important in optimal product pricing and dynamic (or time-varying) pricing. In our experiments, we provide a comparative study including the proposed framework strategies and some other baselines. The accomplished results demonstrate a significant performance for the proposed methods.

## 1. Introduction

Recently, active learning has received a substantial growing interest in literature. With the abundant amounts of unlabeled data, the cost of data labelling is, generally, expensive. Thus, active learning is used for selecting the most informative “beneficial” training samples for the learning model in order to achieve high model accuracy using as few examples as possible [1]. Active learning has proved its superiority in diverse applications such as natural language processing [2] and image processing [3]. The active learning process basically proceeds as follows: first, an initial learning model is trained using a few training samples. Then, additional samples are sequentially added to the training data according to a certain querying strategy. This process repeats until a certain stopping criterion is satisfied [4].

Generally, most of the active learning research mainly focuses on querying data labels to optimize the learning model’s accuracy. Only a few contributions utilize active learning for achieving other objectives. However, in many applications, the data labeling process is costly and the ultimate goal is to optimize a domain-specific objective function, other than minimizing the learning model’s predictive error. Accordingly, in this work, we propose a comprehensive active learning framework which consists of several novel querying strategies for handling general optimization problems where the objective could be some general utility function, not necessarily the learning model’s accuracy. The problem can be framed as selecting the right trade-off for the exploration-exploitation concept. In other words, we encounter a trade-off between minimizing the uncertainty of the target objective function, known as exploration and maximizing the underlying objective function given the available function estimates, which is known as exploitation. The exploration-exploitation trade-off is encountered in machine learning [5] and optimization algorithms [6]. Furthermore, this class of optimization problems experiencing a trade-off between exploration and exploitation is prevalent in many real-world applications of various fields, such as recommender systems [7] and dynamic pricing [8].

In this paper, we provide a comprehensive analysis of active learning from the point of view of the exploration-exploitation trade-off. Our focus is on having a general optimization function, rather than prediction accuracy. For example, the user may like to select a query point that maximizes his profit. As a case study, we consider the application of the proposed active learning framework to some real-world application, namely dynamic pricing for revenue maximization in case of unknown behavior of the customers’ demand [9]. Specifically, firms offering a certain good or service seek to adjust prices in a way that maximizes the obtained revenue. However, the price-demand curve which controls the relation between the price and the corresponding behavior of customers, is usually not known beforehand and has to be inferred. Generally, companies learn the price-demand curve through price experimentation by testing a number of prices and obtaining their corresponding demands from actual selling situations. On the other hand, choosing prices for revealing the price-demand relation could yield revenue losses since such prices are not designed to maximize the achieved revenue [10,11].

Therefore, we are dealing with two conflicting goals: exploration in the form of choosing prices that minimize the uncertainty of the learned demand model and exploitation in the form of setting prices to maximize the objective function, that is, the obtained revenue. The former is accomplished in a framework of active learning: what price should we suggest next to gain the most knowledge of the demand-price function?

The aforementioned problem of revenue maximization with demand learning represents a case study which can be considered an application of our proposed framework. However, the presented active learning framework is general and it can be applied to any objective optimization problem incurring a trade-off between exploration and exploitation.

The proposed active learning framework consists basically of three main active learning approaches: exploration-based, exploitation-based and balancing strategies that handle both exploration and exploitation. For the exploration-based methods, we propose several novel information-theoretic strategies with the aim of minimizing the learning model uncertainty. On the other hand, the exploitation-based methods are designed merely to optimize the target objective function, without taking into consideration the model accuracy. Finally, we present several active learning strategies specifically designed to address the exploration-exploitation trade-off by combining both objectives of optimizing the target objective and obtaining an accurate learning model.

We apply a set of experiments to evaluate the performance of our proposed active learning methods in terms of both aspects: exploitation in terms of the gained utility and exploration by measuring the regression model’s accuracy. In these experiments, we compare the performance of our proposed methods to some standard baselines.

Active learning has been extensively studied in classification problems [4]. However, only few studies investigate applying active learning to regression tasks [12,13,14]. In this work, our presented active learning framework mainly targets regression problems. However, it could be easily adapted to handle classification problems, as well.

Active learning is generally classified into sequential and batch mode settings. In the sequential setting, one query sample is selected per iteration. On the other hand, for the batch mode, a group of samples are simultaneously selected for labeling. In this work, we adopt the sequential active learning approach.

Another scheme used for classifying the active learning methods is based on the query generation process. Specifically, active learning is classified into: pool-based and query synthesis approaches. The pool-based approach is the conventional method which is most commonly used in the active learning literature [4]. In the pool-based scheme, at each iteration, one or more query samples are selected from an unlabeled pool of existing data according to a certain querying criterion and labeling is carried out for these selected samples. On the other hand, the membership query synthesis approach selects one or more synthetic samples from the whole space. In this paper, we apply both approaches—pool-based and query synthesis. Moreover, we perform a comparative study between the two methods. From the experimental results, this work essentially elucidates the significance and the superiority of employing the query synthesis approach over the commonly used pool-based approach. More detailed results will be discussed in Section 7 and Section 8.

The goal of this work is not to provide a group of active learning strategies, instead, we aim to introduce a comprehensive active learning framework including novel strategies, for handling a wide class of objective optimization problems confronting the exploration-exploitation dilemma.

The main contributions of this paper are summarized as follows:

- Provide a comprehensive active learning framework for a general objective optimization, analyzing it from the point of view of the exploration-exploitation trade-off.
- Propose several novel information-theoretic active learning strategies, designed for minimizing the learning model uncertainty.
- Design active learning methods for regression tasks.
- Present a less-myopic active learning method focusing on exploitation or target optimization.
- Develop query synthesis and pool based variants of the proposed active learning strategies and compare the two approaches.
- Apply the proposed active learning framework to a real-world application, namely dynamic pricing with demand learning, as a case study.

The paper is organized as follows: Section 2 presents a literature review. Section 3 presents the problem formulation. Section 4 briefly describes the Bayesian formulation of linear regression model that is applied in our experiments. Then, our proposed active learning strategies are represented in Section 5. After that, Section 7 presents experimental results. Section 8 discusses the main findings. Finally, Section 9 concludes the paper.

## 2. Related Work

In this section, we briefly review the related work.

### 2.1. Active Learning

A comprehensive active learning literature survey can be found in the work by Settles in Reference [4]. Mostly, active learning research is designed to query data samples which enhance the predictive power of the learning model. One of the popular active learning methods is uncertainty sampling [15], which selects a sample that the learning model is most uncertain about. The label uncertainty is often measured using the predictive label variance [16] or the label entropy [17].

Another commonly used active learning strategy is query by committee (QBC) [18]. The QBC strategy hinges on minimizing the version space [4]. A committee of learning models, generally formed using ensemble learning, are trained using the training data acquired so far. Then, the QBC strategy chooses the most controversial data sample, about which the learning models disagree the most. Roy et al. propose an active learning strategy that targets minimizing the generalization error of the learning model [19]. However, their method is computationally intensive.

### 2.2. Active Learning for Regression

Unlike the classification domain, there is limited work that considers active learning for the regression domain. In this work, we mainly focus on regression tasks. However, our proposed active learning framework is general enough and could be applied to classification tasks. In this subsection we briefly overview the main methods of active learning for regression.

Several popular active learning methods have been extended and applied to regression such as query by commitee (QBC) in Reference [13]. In addition, Cai et al. propose an active learning method named, Expected Model Change Maximization (EMCM). Their presented querying method selects the data samples leading to the maximum model change. In their work, they estimate the model change as the gradient of the loss function, typically squared error, with respect to the query sample under consideration.

Wu proposes an active learning approach that considers representativeness and diversity in initial data collection and sequential query selection [14]. The presented approach typically applies k-means clustering to ensure representativeness by choosing data samples that are close to clusters’ centroids. Furthermore, diversity is satisfied by favoring clusters having no labeled data so far. Another work seeking to enhance diversity of data samples is presented in Reference [20].

The pool-based active learning chooses training data points without assuming a prior knowledge of the test distribution. On the other hand, the population-based active learning assumes that the test distribution of data points is known and it seeks to estimate the optimal training input density from which training data points are sampled. Sugiyama et al. develop a population-based active learning approach using weighted least-squares linear regression in Reference [21]. Their proposed method, named ALICE, aims to minimize the conditional expectation of the generalization error given the training data samples.

To our knowledge, applying information-theoretic approaches to active learning for the regression domain is limited, unlike the classification domain. In this work, as demonstrated in Section 5.2, we propose several information-theoretic based active learning querying strategies for regression.

### 2.3. Information-Theoretic Active Learning

In this section, we briefly describe some information-theoretic based active learning methods in literature, that are mainly designed for classification, aiming to enhance the learning model predictive performance.

Guo and Greiner exploit the potential information of the unlabeled data in their proposed active learning strategy [17]. The authors develop their active learning method based on maximizing the mutual information between the underlying query and the conditional labels of the unlabeled pool given the training data. In their method, since the true label is not known, the authors use an optimistic label for the candidate query sample, which is the label leading to the maximum mutual information about the labels of the unlabeled pool samples.

The authors of Reference [22] develop an entropy-based active learning for object recognition. The presented method seeks to minimize the expected entropy of the labels for the unlabeled pool of samples, given the training data acquired so far.

In Reference [23], the authors develop an information-theoretic active learning framework in batch setting mode. Their proposed framework seeks to maximize the mutual information between the candidate sample and the unlabeled pool of samples. The authors propose pessimistic and optimistic approximations of the mutual information by choosing the label minimizing or maximizing the conditional entropy of the labels of the unlabeled samples.

Another information-theoretic metric used in Reference [24], for active learning classification, is the Fisher information ratio (FIR). A major advantage of using the Fisher information metric specifically, is that it accounts for the diversity among the query samples. The proposed method obtains a probability mass function (PMF) over the unlabeled pool by maximizing the FI using semi-definite programming, then the chosen queries are drawn according to the optimized PMF.

In this paper, we propose novel active learning strategies for regression tasks that utilize information-theoretic concepts including: mutual information, Kullback–Leibler divergence and learning model entropy, as described in Section 5.2.

### 2.4. Query Synthesis versus Pool-Based AL

As previously mentioned in Section 1, active learning can be classified into pool-based and query synthesis approaches [4]. The pool-based approach is prevalent in active learning literature, however the query synthesis approach could potentially outperform the pool-based method, since unlike the pool-based, the query sample is chosen from the whole input space and not restricted by a certain pool of samples, that could not be representative for the whole input space or could not contain the optimal query samples. However, the main limitation of the query synthesis approach is that it could not be applicable for tasks requiring human annotation such as image processing and natural language processing, since the synthetically generated samples could be meaningless to the human annotator [4]. Consequently, the query synthesis could mainly be used whenever the query oracle is a scientific experiment or when the underlying input space is continuous such as: the considered dynamic pricing application [8] and some robotics applications [25].

There are a few contributions applying query synthesis for active learning querying. Query synthesis was first introduced in Reference [26]. In Reference [25], the authors approximate the version space by solving a convex optimization problem. Then, the synthetic query is generated by extracting the principal component that would shrink the version space.

In this work, we implement our proposed active learning strategies in both ways: query synthesis and pool-based. The experimental results indicate that the query synthesis approach has superior performance compared to the pool-based approach as discussed in Section 7. This is intuitively logical because the query synthesis approach optimizes the query criteria over the whole input space, so the returned solution is optimal since it is not restricted to be in a certain pool of samples. Moreover, the query synthesis approach is significantly more computationally efficient than the pool-based approach since for each iteration, the former optimizes the underlying query strategy one time, while the latter evaluates the querying strategy over all the pool samples and chooses the best sample of them to query, which is computationally intensive, especially that the pool size (the number of available unlabeled samples) is used to be large.

### 2.5. Active Learning for Objective Optimization

As mentioned in the introduction, Section 1 and as discussed so far, most of the active learning work in literature aims to enhance the predictive accuracy of the learning model. There are only limited research contributions that use active learning for achieving general real-world objectives other than the model predictive power. In this section, we discuss the main contributions that utilize active learning for achieving a general objective other than the learning model accuracy.

Saar and Provost design an active learning querying method named Goal-Oriented Active Learning (GOAL) for decision making. In their paper, the authors apply their proposed active learning method to customer targeting campaigns [27]. They typically consider binary decision (classification) problem, which is whether to target a specific customer or not, given that customer targeting incurs some cost. Their proposed method queries data samples that are close to decision threshold to enhance decision learning. However, the GOAL method does not consider the trade-off between learning optimal decisions and profit maximization.

Garnett et al. adopt active learning for two binary classification problems, active search and active surveying in Reference [28]. The authors utilize the Bayesian decision theory and they propose less-myopic approximations to the optimal policy by considering multiple step look-ahead of the underlying utility functions of both problems.

Marcela et al. develop an active learning approach for solving multi-objective optimization, named ε-Pareto Active Learning (ε-PAL) [29]. Their approach assumes that the considered objectives follow a Gaussian process distribution, so they use Bayesian optimization framework. However, their work does not focus on the exploration-exploitation trade-off that may exist among the underlying different objectives, which is the main concern of our presented work.

Another active learning scheme for sequential decision making is the knowledge-gradient (KG) method [30]. The KG method is an exploitation-based strategy that considers several alternatives and chooses the alternative maximizing the expected improvement of a certain utility function. The knowledge-gradient method maintains a Bayesian predictive distribution for each alternative’s utility and these posterior distributions are updated upon acquiring new observations. However, the KG method could be computationally expensive for large number of alternatives.

Unlike the KG method, our framework considers the distribution of a certain utility function that is evaluated using a learning model, specifically the Bayesian linear regression. Another difference between the KG method and our proposed methods is that the KG method is inherently designed in pool-based setting where the selection is performed from a finite set of alternatives. On the other hand, our proposed approaches are general to be applied in pool-based or query synthesis setting as indicated in Section 5.1. In addition, the KG method is a pure exploitation method that does not explicitly consider exploration. However, in this work, we provide several less-myopic methods balancing between exploitation and exploration described in Section 5.4.

The mean objective cost of uncertainty (MOCU) method proposed in References [31,32] handles model uncertainty in a novel way. The MOCU method essentially studies the impact of the model uncertainty on performance degradation in terms of some incurred cost. Specifically, the MOCU criterion evaluates model uncertainty by measuring the differential cost between the current estimated model and the optimal model which minimizes the expected cost.

### 2.6. The Exploration-Exploitation Trade-Off

The exploration and exploitation trade-off has been extensively studied in many contexts including: reinforcement learning [5], multi-armed bandit problems [33] and evolutionary optimization [6].

Krause and Guestrin handle the trade-off between exploration and exploitation in their active learning method for handling spatial phenomena such as river monitoring [34]. The authors use Gaussian Processes (GPs) in their model, with unknown kernel parameters. They propose a non-myopic active learning approach for handling the trade-off between exploration, which aims to decrease the uncertainty about the model parameters and exploitation, which seeks to near-optimal observations using the estimated model parameters so far. However, they use static split between exploration and exploitation as two separate phases and they derive some bounds for the length of the exploration phase. On the other hand, our proposed methods described in Section 5.4 make probabilistic transitions/balance between exploration and exploitation. A dynamic balance between exploration and exploitation that is performed probabilistically could be more powerful than static balance, especially for real world applications that could have noisy observations. In such a case it is hard to predict a predefined period of exploration.

The multi-armed bandit (MAB) context is a class of sequential decision making problems originally developed in Reference [35]. The objective is to maximize rewards but under uncertainty and incomplete feedback about rewards, so there is a trade-off between performing an action that gathers information regarding reward (exploration) and making a decision that maximizes the immediate reward given the information gathered so far (exploitation). In our experiments, we apply the upper confidence bound algorithm (UCB) [36], a popular algorithm developed in the context of MAB, as a baseline to compare with.

Although the primary objective of reinforcement learning is to maximize the cumulative rewards, which is typically exploitation, exploration plays a significant role in reinforcement learning as demonstrated in Reference [5], since without exploration, the agent could simply derive sub-optimal plans. So, achieving the balance between exploration and exploitation is the core issue in reinforcement learning. However, reinforcement learning is generally computationally expensive, so it is not scalable for large state spaces. Furthermore, reinforcement learning requires a considerable amount of training data, unlike active learning which is designed for limited data requirements. The work of Reference [37] relates the concept of exploration-exploitation trade-off with bias-variance trade-off.

The exploration-exploitation trade-off has been extensively addressed in the context of evolutionary optimization. In such context, exploration is defined as visiting new regions of the search space, while exploitation denotes visiting regions of the search space within the neighborhood of previously explored points. A comprehensive review of the exploration-exploitation trade-off in evloutionary optimization is presented in Reference [6].

## 3. Problem Formulation

As mentioned in the introduction, this work focuses on regression tasks since it is prominent in different applications such as energy consumption prediction [38] and price-demand elasticity estimation [8,39]. Specifically, in this work, we apply linear regression model but our proposed active learning framework is general and can be applied to any other regression model. Furthermore, the proposed strategies can be adapted to classification models as well.

We consider the following linear regression problem:

(1) $$y=\beta^{T}x+\epsilon$$

where *x* is the input feature vector such that $x\in R^{d}$, where *d* is the dimensionality of the feature vector, *y* denotes the regression response variable y∈R and ϵ is a random error term such that $\epsilon ∼N(0,\sigma^{2})$ and $\beta \in R^{d}$ denotes the regression model coefficients.

This work particularly tackles the class of optimization problems which have a certain utility function *u* to be optimized, for any regression task. However, the utility function *u* incurs some uncertainty which can be estimated using a probabilistic regression model. Such problems pose the challenging problem of how to strike a balance between maximizing the objective function *u* (exploitation) and minimizing the uncertainty about the utility function (exploration). In this work, we develop a novel active learning framework consisting of various strategies to interactively seek a balance between exploitation and exploration.

### Notation

In this section, we introduce the adopted notation used in the proposed active learning framework.

First, the training data acquired so far is denoted as $D={\left\{(x_{i},y_{i})\right\}}_{i=1}^{N}$, the training data term D is expressed in terms of a set of pairs of input data samples $x_{i}$ and their corresponding labels $y_{i}$, where *N* is the number of data samples acquired so far.

The matrix of input data points is denoted as $X\in R^{N\times d}$, such that each row *x* represents one data sample and *d* is the dimensionality of the data point *x*. For $Y\in R^{N\times 1}$, it represents the vector of the corresponding output variables. The matrix of data samples whose outputs require prediction is denoted as $X^{*}$, such that $X^{*}\in R^{m\times d}$, where *m* is the size of data samples to be predicted. In addition, $Y^{*}$ represents the vector of the corresponding output variables and $Y\in R^{m\times 1}$. Similarly, in case of predicting a single data point, the data sample is denoted as $x^{*}$ and $y^{*}$ is its corresponding output.

In the adopted linear regression algorithm described in Section 4, the regression coefficients are denoted as β. In addition, $\mu_{\beta }$ and $\Sigma_{\beta }$ are the mean and covariance matrix of β, respectively.

In the proposed active learning framework, U denotes the unlabeled pool of data samples and $Y_{U}$ represents the responses of the samples in the pool. The utility function *u* represents the objective function to be optimized using active learning as defined in Section 5.3 and Section 5.4.

## 4. Preliminaries: Bayesian Linear Regression

In this section, we briefly describe the Bayesian linear regression model used in the proposed active learning framework. We adopt the Bayesian linear regression model due to several reasons. First, the class of optimization problems that we handle involves uncertainty of the utility function, which can be estimated using probabilistic regression models such as the Bayesian linear regression. Moreover, most active learning querying strategies depend on the uncertainty of predictions, so it is compelling that we use a regression model providing not only predictions but also uncertainty of the obtained predictions and Bayesian linear regression provides such information. Finally, in active learning settings, the initial data points available for training is essentially limited which could result in over-fitting, especially for noisy data, so applying Bayesian linear regression helps to combat the potential over-fitting.

The underlying regression problem is formulated as indicated in Equation (1), in Section 1. According to Equation (1), we have two major parameters in the regression model, the regression model coefficients β and the noise variance $\sigma^{2}$, so we adopt Bayesian linear regression with conjugate prior of $\left(\beta ,\sigma^{2}\right)$.

Since the noise variance parameter $\sigma^{2}$ is a key parameter in the model and we have some prior knowledge about it, for example it must be positive, we can use a conjugate prior distribution for both parameters β and $\sigma^{2}$. We assume an Inverse Gamma prior distribution for $\sigma^{2}$, $\sigma^{2}∼\mathrm{IG}\left(a_{\sigma },b_{\sigma }\right)$.

(2) $$p\left(\sigma^{2}\right)=\frac{{\left(b_{\sigma }\right)}^{a_{\sigma }}}{\Gamma (a_{\sigma })}\sigma^{-2(a_{\sigma }+1)}e^{-\frac{b_{\sigma }}{\sigma^{2}}}$$

where $a_{\sigma }>1,b_{\sigma }>0$ and $\sigma^{2}>0$. The conjugate prior $p(\beta ,\sigma^{2})$ can be expressed as a Normal Inverse Gamma (NIG) distribution as follows:

(3) $$p\left(\beta ,\sigma^{2}\right)=p\left(\beta |\sigma^{2}\right)p\left(\sigma^{2}\right)=N\left(\mu ,\sigma^{2}\Sigma \right)\mathrm{IG}\left(a_{\sigma },b_{\sigma }\right)=\mathrm{NIG}\left(\mu ,\Sigma ,a_{\sigma },b_{\sigma }\right)$$

- Conjugate Posterior Distribution:
  According to Reference [40], the conjugate posterior distribution $p(\beta ,\sigma^{2}|D)$ is a Normal Inverse Gamma (NIG) distribution as follows:
  (4) $$p\left(\beta ,\sigma^{2}|D\right)=\mathrm{NIG}\left(\mu_{\beta |D},\Sigma_{\beta |D},a_{\sigma |D},b_{\sigma |D}\right)$$
  Let μ and Σ be the prior expectation and covariance matrix of parameters β, respectively. The posterior mean $\mu_{\beta |D}$ is evaluated as follows:
  (5) $$\mu_{\beta |D}={\left(X^{T}X+\Sigma^{-1}\right)}^{-1}\left(\Sigma^{-1}\mu +X^{T}y\right)$$
  The posterior covariance $\Sigma_{\beta |D}$ is calculated as follows:
  (6) $$\Sigma_{\beta |D}={\left(X^{T}X+\Sigma^{-1}\right)}^{-1}$$
  The posterior updates of noise distribution parameters $a_{\sigma }$ and $b_{\sigma }$ parameters are given by:
  (7) $$a_{\sigma |D}=a_{\sigma }+\frac{N}{2}$$
  (8) $$b_{\sigma |D}=b_{\sigma }+\frac{1}{2}\left(y^{T}y+\mu^{T}\Sigma^{-1}\mu -\mu_{\beta |D}^{T}{\Sigma_{\beta |D}}^{-1}\mu_{\beta |D}\right)$$
  As derived in Reference [41], the marginal posterior distribution for β, denoted as β|D, is a multivariate Student-t distribution as follows:
  (9) $$\beta |D∼t_{2a_{\sigma |D}}\left(\mu_{\beta |D},\frac{b_{\sigma |D}}{a_{\sigma |D}}\Sigma_{\beta |D}\right)$$
  For a random variable *Z* that follows a multivariate Student-T distribution, defined as $t_{v}\left(\mu_{0},\Sigma_{0}\right)$, the expectation and the covariance matrix of *Z* are calculated, respectively, as follows:
  (10) $$E\left[Z\right]=\mu_{0}$$
  (11) $$\Sigma_{Z}=\frac{v}{v-2}\Sigma_{0}$$
  where *v* is the number of degrees of freedom for the Student-T distribution $t_{v}\left(\mu ,\Sigma \right)$.
  According to Equations (9), (18) and (11), the expectation and the covariance matrix of the marginal β|D distribution are evaluated as follows:
  (12) $$E\left[\beta |D\right]=\mu_{\beta |D}$$
  (13) $$Cov[\beta |D]=\frac{b_{\sigma |D}}{a_{\sigma |D-1}}\Sigma_{\beta |D}$$
- Posterior predictive distribution of *Y*:
  As derived in Reference [40], the posterior predictive distribution p(Y|D) is evaluated as follows:
  (14) $$\begin{array}{cc} p(Y|D) & =\int p\left(Y|\beta ,\sigma^{2}\right)p\left(\beta ,\sigma^{2}|D\right) \end{array}$$
  (15) $$\begin{array}{c} =N\left(X\beta ,\sigma^{2}I_{m}\right)\times NIG\left(\mu_{\beta |D},\Sigma_{\beta |D},a_{\sigma |D},b_{\sigma |D}\right) \end{array}$$
  (16) $$\begin{array}{c} =t_{2a_{\sigma |D}}\left(X\mu_{\beta |D},\frac{b_{\sigma |D}}{a_{\sigma |D}}\left(I_{m}+X\Sigma_{\beta |D}X^{T}\right)\right) \end{array}$$
  To predict a vector of output responses $Y^{*}$, corresponding to a matrix of data points $X^{*}$, the posterior predictive distribution of the output vector $Y^{*}$ is defined as follows:
  (17) $$p\left(Y^{*}|X^{*},D\right)∼t_{2a_{\sigma |D}}\left(E\left[Y^{*}|X^{*},D\right],A_{Y^{*}|X^{*},D}\right)$$
  The posterior expectation of the predicted responses $Y^{*}$ is calculated as:
  (18) $$E\left[Y^{*}|X^{*},D\right]=X^{*}\mu_{\beta |D}$$
  where $A_{Y^{*}|X^{*},D}$ is calculated as:
  (19) $$A_{Y^{*}|X^{*},D}=\frac{b_{\sigma |D}}{a_{\sigma |D}}\left(I_{m}+X^{*}\Sigma_{\beta |D}{X^{*}}^{T}\right)$$
  However, the covariance matrix for a multivariate Student-T distribution $t_{v}\left(\mu ,A\right)$ is estimated as:
  (20) $$\Sigma =\frac{v}{v-2}A$$
  Consequently, from Equation (17) and substituting from Equation (19) into Equation (20), the posterior variance of the predicted responses $Y^{*}$ is calculated as:
  (21) $$\Sigma_{Y^{*}|X^{*},D}=\frac{b_{\sigma |D}}{a_{\sigma |D}-1}\left(I_{m}+X^{*}\Sigma_{\beta |D}{X^{*}}^{T}\right)$$
  To predict a single label $y^{*}$, the predictive posterior distribution $p(y^{*}|x^{*},D)$ is evaluated as:
  (22) $$p\left(y^{*}|x^{*},D\right)∼t_{2a_{\sigma |D\cup (x^{*},y^{*})}}\left(E\left[y^{*}|x^{*},D\right],\sigma_{y^{*}|x,D}\right)$$
  According to Equations (17) and (18), the posterior expectation of the predicted label $y^{*}$ is calculated as:
  (23) $$E\left[y^{*}|x^{*},D\right]={x^{*}}^{T}\mu_{\beta |D}$$
  Similarly, using Equations (17) and (21), the posterior variance of the predicted value $y^{*}$ is defined as:
  (24) $$\sigma_{y^{*}|x^{*},D}^{2}=\frac{b_{\sigma |D}}{a_{\sigma |D}-1}\left(1+{x^{*}}^{T}\Sigma_{\beta |D}x^{*}\right)$$

In this section, we have provided the final formulations for Bayesian linear regression model. The interested readers can find more details in References [40,42].

## 5. Proposed Active Learning Framework

In this section, we present our proposed active learning framework for handling optimization problems, encountering an exploration-exploitation trade-off.

**Figure 1:**
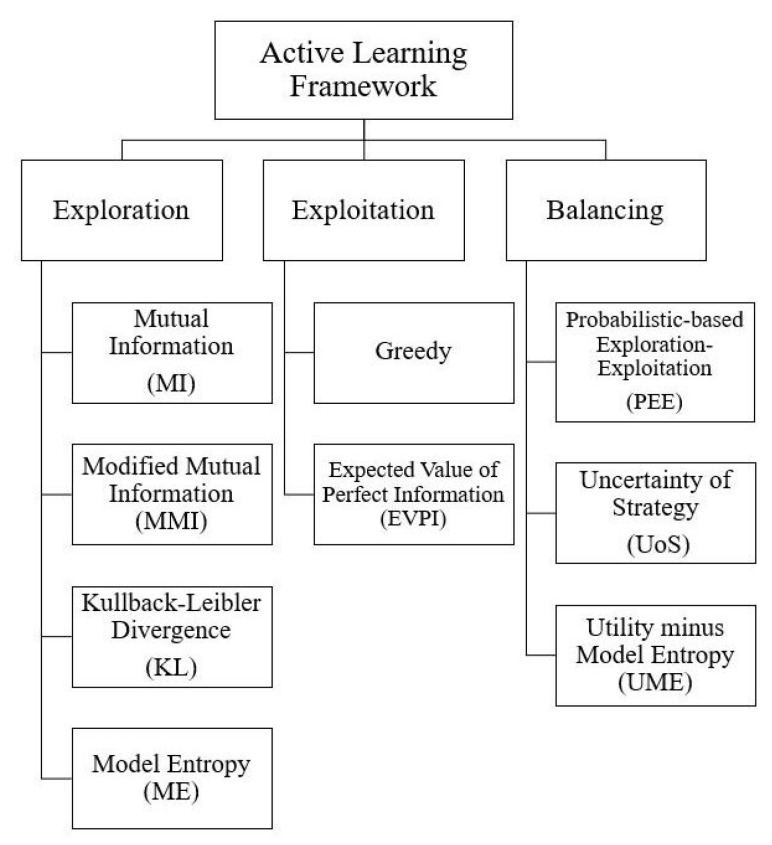
The Proposed Active Learning Framework.

First, we describe the general active learning settings. Then, we introduce our proposed active learning strategies which are mainly classified into: exploration-based, exploitation-based and strategies that balance exploration and exploitation. Figure 1 shows the proposed active learning framework.

### 5.1. Active Learning Schemes

Active learning can be applied in different modes that define how a new query point is generated. We describe three different schemes, the first two methods are generally known in literature and we define the third one because we incorporate it into some of our proposed strategies.

- Pool-based
  This is the conventional approach that is mostly used in the active learning literature. In the pool-based approach, there exists an unlabeled pool of data samples $X_{U}$ and at each iteration, one or more query example(s) $x^{*}$ is selected from the pool according to a certain querying criterion. Algorithm 1 describes the pool-based active learning approach.
- Membership Query Synthesis
  Unlike the pool-based approach, the membership query synthesis scheme is not commonly used in the active learning literature. In contrast with the pool-based active learning, the membership query synthesis does not select data samples out of a certain pool of unlabeled data. Alternatively, this approach essentially generates and queries synthetic data samples of the entire input space. Algorithm 2 explains the query synthesis approach.
  This approach is very efficient and is not computationally intensive compared to the pool-based approach. The reason for the query synthesis’s computational efficiency is that instead of iterating over the large unlabeled pool of samples and evaluating a certain selection criterion such as mutual information, the query synthesis approach directly generates a synthetic data sample to achieve a certain objective. For example, our proposed query synthesis approach optimizes the underlying querying metric using optimization algorithms. The query synthesis approach is not only computationally efficient, it could be more compelling than the pool-based approach since the generated query sample is not restricted to be part of an unlabeled pool, so the synthetically generated query sample could be more informative and beneficial than the examples in the pool.
- Membership Query Synthesis without a Predefined Pool
  The query synthesis approach does not need to have a pool of samples. However, some active learning strategies exploit the potential information in the unlabeled data to guide the sample selection such as mutual information strategy defined subsequently in Equation (28) and the KL divergence strategy defined in Equation (47). Consequently, such strategies rely on the existence of some unlabeled data to estimate how useful or how representative a certain query point is. However, for some applications, the unlabeled data could not exist or if they exist, they may not be a representative sample for the input space. In such cases, one could generate a representative and diverse sample of unlabeled data using the domain knowledge of the feature space. Another way for generating unlabeled representative data could be to apply any reasonable clustering algorithm using the available training data and the cluster centroids can be used as representatives of the unobserved data. Algorithm 3 elucidates this approach.

**Algorithm 1** Pool-based Active Learning
**Input:** A dataset $D={\left\{(x_{j},y_{j})\right\}}_{j=1}^{N}$, a general active learning strategy *S*, a utility function *u*, number of iterations *T*, a discount factor γ and a generation method for creating synthetic queries GenerateQueryPoint().**Output:** A Learned model $\theta_{T}$ and a cumulative gained utility $u_{T}$.$D_{L}\leftarrow$$N_{init}$ labeled data samples randomly chosen out of D.Train the regression model using the initial training data to obtain initial model $\theta_{0}$.
$D_{U}\leftarrow D\backslash D_{L}$

**repeat**
 **for each** $x_{k}\in D_{U}$ **do**  $S(x_{k})\leftarrow$ Apply a certain active learning strategy *S* to $x_{k}$, using current model estimate $\theta_{i}$. **end for** $x^{*}={arg max}_{x_{k}}S\left(x_{k}\right)\;\;\forall_{k},\;\;k\in \left\{1\ldots \right|D_{U}\left|\right\}$. $y^{*}\leftarrow$ the true label for the query sample $x^{*}$. Add the acquired data point $\left(x^{*},y^{*}\right)$ to the training data: $D_{L}\leftarrow D_{L}\cup \left(x^{*},y^{*}\right)$. Evaluate the utility $u_{i}$ using the new acquired point: $u_{i}\leftarrow u\left(x^{*},y^{*}\right)$. Update the regression model $\theta_{i}$ using the new acquired point $\left(x^{*},y^{*}\right)$.**until***T* iterations executed**return** The learned model $\theta_{T}$ and the cumulative discounted utility $u_{T}=\sum_{i=1}^{T}\gamma^{i-1}u_{i}$.


In our experiments, we develop several novel active learning strategies and apply them in the pool-based and query synthesis schemes. For the strategies that use the unlabeled data samples for guiding its selection such as mutual information (MI), modified mutual information (MMI) and Kullback–Leibler divergence (KL), we apply the three aforementioned schemes. More details are provided in the experiments section, Section 7.

**Algorithm 2** Query Synthesis Active Learning
**Input:** A dataset $D={\left\{(x_{j},y_{j})\right\}}_{j=1}^{N}$, a general active learning strategy *S*, a utility function *u*, number of iterations *T*, a discount factor γ and a generation method for creating synthetic queries GenerateQueryPoint().**Output:** A Learned model $\theta_{T}$ and a cumulative gained utility $u_{T}$.$D_{L}\leftarrow$$N_{init}$ labeled data samples randomly chosen out of D.Train the regression model using the initial training data to obtain initial model $\theta_{0}$.
**repeat**
 $x^{*}=GenerateQueryPoint\left(S,D_{U},\theta_{i}\right)$. $y^{*}\leftarrow$ the true label for the query sample $x^{*}$. Add the acquired data point $\left(x^{*},y^{*}\right)$ to the training data: $D_{L}\leftarrow D_{L}\cup \left(x^{*},y^{*}\right)$. Evaluate the utility $u_{i}$ using the new acquired point: $u_{i}\leftarrow u\left(x^{*},y^{*}\right)$. Update the regression model $\theta_{i}$ using the new acquired point $\left(x^{*},y^{*}\right)$.**until***T* iterations executed **return** The learned model $\theta_{T}$ and the cumulative discounted utility $u_{T}=\sum_{i=1}^{T}\gamma^{i-1}u_{i}$.


**Algorithm 3** Query Synthesis Active Learning without a predefined pool
**Input:** A small dataset of $N_{init}$ points $D={\left\{(x_{j},y_{j})\right\}}_{j=1}^{N_{init}}$, a general active learning strategy *S*, a utility function *u*, number of iterations *T*, a discount factor γ and a generation method for creating synthetic queries GenerateQueryPoint().**Output:** A Learned model $\theta_{T}$ and a cumulative gained utility *u*.$D_{L}\leftarrow N_{init}$ labeled data samples randomly chosen out of D.Train the regression model using the initial training data to obtain initial model $\theta_{0}$.U← Construct a representative sample of unlabeled data using for example, domain knowledge or clustering.
**repeat**
  $x^{*}=GenerateQueryPoint\left(S,U,\theta_{i}\right)$. $y^{*}\leftarrow$ the true label for the query sample $x^{*}$. Add the acquired data point $\left(x^{*},y^{*}\right)$ to the training data: $D_{L}\leftarrow D_{L}\cup \left(x^{*},y^{*}\right)$. Evaluate the utility $u_{i}$ using the new acquired point: $u_{i}\leftarrow u\left(x^{*},y^{*}\right)$. Update the regression model $\theta_{i}$ using the new acquired point $\left(x^{*},y^{*}\right)$.**until***T* iterations executed**return** The learned model $\theta_{T}$ and the cumulative discounted utility $u_{T}=\sum_{i=1}^{T}\gamma^{i-1}u_{i}$.


### 5.2. Exploration-Based Strategies

In this section, we describe our novel proposed exploration-based active learning strategies for regression. The exploration-based strategies mainly target enhancing the regression model predictive performance. The presented strategies are not limited to a certain application or a class of problems, they are quite general and could be applied in any settings where the objective is to boost the regression model accuracy. The most popular active learning methods such as uncertain sampling [15] and Query by Committee [18] seek to query the most “uncertain” sample, that is, the data sample about which the learning model is the most uncertain. Although this seems helpful for the learning model either classification or regression, the uncertain sampling approach does not consider the potential information of the unlabeled pool of examples. Thus, the uncertain sampling could select noisy patterns or outliers. On the other hand, querying samples not only based on the query sample but also on the unlabeled samples of the pool ([17,23]) is more promising since such approach is less myopic and it utilizes the information of the plentiful unlabeled pool.

The following proposed exploration strategies are mainly based on information theory [43]. To our knowledge, it is the first time that information theoretical concepts (such as mutual information, Kullback–Leibler divergence and model entropy) are applied in active learning for regression. Some information-theoretic metrics such as predictive label entropy, Fisher information and mutual information have been employed for active learning in classification problems [17,22,23]. However, such information theoretic metrics have not been considered yet for regression problems.

Depending solely on a single query sample information could lead to choosing noisy samples or outliers [19]. It is well-known that an outlier does more damage than help. Consequently, our proposed exploration-based active learning strategies exploit the potential information existing in the unlabeled pool of samples and the learning model uncertainty. Moreover, incorporating the information of the unlabeled pool such as mutual information, into the selection strategy, advocates querying representative samples.

#### 5.2.1. Mutual Information (MI)

The mutual information criterion aims to query the sample $x^{*}$ which effectively holds a substantial amount of information about the labels of the unlabeled pool. Thus, this strategy chooses the sample $x^{*}$ that maximizes the mutual information between its label $y^{*}$ and the labels of the remaining unlabeled samples of the pool excluding $x^{*}$, denoted as $Y_{U}$.

The mutual information between the query sample $x^{*}$ and the labels of the unlabeled pool $Y_{U}$ is defined as:

(25) $$I\left(x^{*},Y_{U}\right)=H\left(Y_{U}|D\right)-H\left(Y_{U}|x^{*},D\right)$$

where D denotes the labeled training data acquired so far.

The first term $H(Y_{U}|D)$ represents the prior entropy (or uncertainty) of all the labels of the unlabeled pool of samples. Similarly, the second term $H(Y_{U}|x^{*},D)$ denotes the entropy of the labels of unlabeled pool of samples but after acquiring the new query point $x^{*}$. From Equation (25), it can be noted that maximizing $I(x^{*},Y_{U})$ is equivalent to minimizing the conditional entropy $H(Y_{U}|x^{*},D)$, which is defined as follows:

(26) $$H\left(Y_{U}|x^{*},D\right)=\int_{y^{*}}p\left(y^{*}|x^{*},D\right)H\left(Y_{U}|x^{*},y^{*},D\right)\;dy^{*}$$

To simplify computations, Equation (26) could be approximated by eliminating the integration over all the possible labels of $y^{*}$ and using the expected value of it $E[y^{*}]$. Other approximations are made in literature [22,23], using the optimistic or the pessimistic label. However, we found that employing the expectation could be more reasonable. Accordingly:

(27) $$H\left(Y_{U}|x^{*},D\right)=H\left(Y_{U}|\left(x^{*},y^{*}\right),D\right)$$

where $y^{*}$ is the expected predicted label of the data point $x^{*}$, which is calculated using Equation (23).

(28) $$H\left(Y_{U}|\left(x^{*},y^{*}\right),D\right)=\int_{Y}p\left(Y|X,D\cup \left(x^{*},y^{*}\right)\right)\log(p\left(Y|X,D\cup \left(x^{*},y^{*}\right)\right)\;dY$$

As mentioned in Section 4, the posterior predictive distribution of the predictive labels vector *Y*, $p(Y|X,D\cup \left(x^{*},y^{*}\right))$ is a multivariate Student-T distribution which is defined as follows:

(29) $$p\left(Y|X,D\cup \left(x^{*},y^{*}\right)\right)=t_{2a_{\sigma |D\cup (x^{*},y^{*})}}\left(Y,E\left[Y|X,D\cup \left(x^{*},y^{*}\right)\right],\Sigma_{Y|X,D\cup (x^{*},y^{*})}\right)$$

The posterior expectation $E[Y|X,D\cup \left(x^{*},y^{*}\right)]$ and the covariance matrix $\Sigma_{Y|X,D\cup (x^{*},y^{*})}$ are evaluated using the Bayesian linear regression model formulations described in Section 4, using Equations (18) and (21), respectively. However, this method and all our proposed methods are general and can be applied using any regression model that provides uncertainty of its predictions.

According to Reference [41], the final formulation of the entropy of a random variable *Z* following a Student-t distribution $t_{v_{Z}}$ is given by:

(30) $$H\left(Z\right)=\frac{1}{2}\log det\left(R_{Z}\right)+\phi \left(v_{Z},d\right)+\frac{v_{Z}+d}{2}M\left(v_{Z},d,\Delta \right)$$

where $R_{Z}$ denotes the correlation matrix of *Z*, *d* is the dimensionality of *Z* and $v_{Z}$ represents the number of degrees of freedom for the Student-t distribution. In addition, $\phi (v_{Z},d)$ is a constant depending on $v_{Z}$ and *d* and $\Delta_{Z}={{\mu_{Z}}^{*}}^{T}{{R_{Z}}^{*}}^{-1}{\mu_{Z}}^{*}$.

(31) $$M\left(a_{Z},d,\Delta \right)=e^{-\frac{\Delta_{Z}}{2}}\sum_{j=0}^{\infty }\frac{1}{j!}\left\{\Psi \left(\frac{d+v_{Z}+2j}{2}\right)-\Psi \left(\frac{v_{Z}}{2}\right)\right\}$$

where Ψ is the digamma function which is defined as:

(32) $$\Psi \left(x\right)=\frac{d}{dx}\ln\left(\Gamma \left(x\right)\right)$$

Accordingly, the conditional entropy of $Y_{U}$, $H(Y_{U}|\left(x^{*},y^{*}\right))$, is calculated using Equation (30) as follows:

(33) $$H\left(Y_{U}|\left(x^{*},y^{*}\right),D\right)=\frac{1}{2}\log det\left({R_{Y}}^{*}\right)+\phi \left(2a^{*},m\right)+\frac{2a^{*}+m}{2}M\left(2a^{*},m,\Delta_{Y}\right)$$

where *m* is the number of data points to be predicted, that is, it is the length of the predicted output vector $Y_{U}$. To simplify notation, let $a^{*}=a_{\sigma |D\cup (x^{*},y^{*})}$, ${\mu_{Y}}^{*}=\mu_{Y|D\cup (x^{*},y^{*})}$, ${\Sigma_{Y}}^{*}=\Sigma_{Y|D\cup (x^{*},y^{*})}$ and ${R_{Y}}^{*}=R_{Y|D\cup (x^{*},y^{*})}$. For $\Delta_{Y}$, it is evaluated as follows:

(34) $$\Delta_{Y}={{\mu_{Y}}^{*}}^{T}{{R_{Y}}^{*}}^{-1}{\mu_{Y}}^{*}$$

such that ${R_{Y}}^{*}$ denotes the correlation matrix of the unlabeled samples *Y* after acquiring the query sample $x^{*}$.

The term $M(2a^{*},m,\Delta_{Y})$ can be evaluated using Equation (31):

(35) $$M\left(2a^{*},m,\Delta_{Y}\right)=e^{-\frac{{{\mu_{Y}}^{*}}^{T}{{R_{Y}}^{*}}^{-1}{\mu_{Y}}^{*}}{2}}\sum_{j=0}^{\infty }\frac{1}{j!}\left\{\Psi \left(\frac{m+2a^{*}+2j}{2}\right)-\Psi \left(a^{*}\right)\right\}$$

Using algebraic manipulations, the summation in Equation (35) converges as follows:

(36) $$\sum_{j=0}^{\infty }\frac{1}{j!}\left\{\Psi \left(\frac{m+a^{*}+2j}{2}\right)-\Psi \left(\frac{a^{*}}{2}\right)\right\}=e\times \left(\Psi \left(a^{*}+\frac{m}{2}+1\right))-\Psi \left(a^{*}\right)\right)$$

Accordingly, substituting from Equation (36) into Equation (35) results in:

(37) $$M\left(2a^{*},d,\Delta_{Y}\right)=\left(\Psi \left(a^{*}+\frac{m}{2}+1\right))-\Psi \left(a^{*}\right)\right)\times e^{-\frac{{{\mu_{Y}}^{*}}^{T}{{R_{Y}}^{*}}^{-1}{\mu_{Y}}^{*}}{2}+1}$$

Then, after substituting from Equation (37) into Equation (33), the conditional entropy $H(Y_{U}|\left(x^{*},y^{*}\right),D)$ can be evaluated as:

(38) $$H\left(Y_{U}|\left(x^{*},y^{*}\right),D\right)=\frac{1}{2}\log det\left(R_{Y}^{*}\right)+\phi \left(2a^{*},m\right)+\left(2a^{*}+\frac{m}{2}\right)\left(\Psi \left(a^{*}+\frac{m}{2}+1\right))-\Psi \left(a^{*}\right)\right)\times e^{-\frac{{{\mu_{Y}}^{*}}^{T}{{R_{Y}}^{*}}^{-1}{\mu_{Y}}^{*}}{2}+1}$$

Finally, the query sample $x^{*}$ that maximizes the mutual information essentially minimizes the conditional entropy of the unlabeled pool of samples as indicated in Equation (25). Consequently, the query sample $x^{*}$ minimizing the conditional entropy $H(Y_{U}|\left(x^{*},y^{*}\right),D)$ can be evaluated as follows:

(39) $$\begin{array}{c} x_{MI}={arg min}_{x^{*}}\frac{1}{2}\log det\left(R_{Y|D\cup (x^{*},y^{*})}\right)+\phi \left(2a_{\sigma |D,\cup (x^{*},y^{*})},m\right)+\left(a_{\sigma |D,\cup (x^{*},y^{*})}+\frac{m}{2}\right)\times \\ \left(\Psi \left(a_{\sigma |D,\cup (x^{*},y^{*})}+\frac{m}{2}+1\right))-\Psi \left(a_{\sigma |D,\cup (x^{*},y^{*})}\right)\right)e^{-\frac{\mu_{Y}^{T}|D,\cup \left(x^{*},y^{*}\right)R_{Y|D,\cup (x^{*},y^{*})}^{-1}\mu_{Y|D,\cup (x^{*},y^{*})}}{2}+1} \end{array}$$

Simplifying Equation (39) by eliminating the term $\phi (2a_{\sigma |D,\cup (x^{*},y^{*})},m)$ since it is a constant that does not depend on the query sample, because $a_{\sigma |D,\cup (x^{*},y^{*})}$ basically depends on the number of data being observed, as indicated in Equation (7). Thus:

(40) $$\begin{array}{c} x_{MI}={arg min}_{x^{*}}\frac{1}{2}\log det\left(R_{Y|D\cup (x^{*},y^{*})}\right)+\left(a_{\sigma |D,\cup (x^{*},y^{*})}+\frac{m}{2}\right)(\Psi \left(a_{\sigma |D,\cup (x^{*},y^{*})}+\frac{m}{2}+1\right))\\ -\Psi \left(a_{\sigma |D,\cup (x^{*},y^{*})}\right))\times e^{-\frac{\mu_{Y|D,\cup (x^{*},y^{*})}^{T}R_{Y|D,\cup (x^{*},y^{*})}^{-1}\mu_{Y|D,\cup (x^{*},y^{*})}}{2}+1} \end{array}$$

For computational efficiency purposes, we evaluate the log determinant of the correlation matrix $R_{Y}$ and its inverse using Cholesky decomposition since the correlation matrix is a symmetric positive semi-definite matrix.

We apply three variants of this active learning strategy: pool-based, query synthesis and query synthesis without pool, which are described in Section 5.1.

#### 5.2.2. Modified Mutual Information (MMI)

The modified mutual information strategy is basically akin to the aforementioned strategy. This method maximizes the mutual information defined in Equation (25) but it evaluates the first term of that equation, $H(Y_{U}|D)$, which represents the entropy of the labels of the unlabeled samples and does not ignore it. The intuition of this querying strategy is to account for the impact of the query sample $\left(x^{*},y^{*}\right)$ on reducing the joint entropy of the unlabeled samples $H(Y_{U}|D)$. In other words, if the first term is ignored and we just focus on minimizing the conditional entropy given the underlying query sample $H(Y_{U}|\left(x^{*},y^{*}\right),D)$, we may choose a sample $x^{*}$ that is redundant and not informative in case the entropy before acquiring $x^{*}$, $H(Y_{U}|D)$ is inherently negligible.

Accordingly, the modified mutual information equation is defined using Equation (25) but without ignoring the first term.

(41) $$I\left(x^{*},Y_{U}\right)=H\left(Y_{U}|D\right)-H\left(Y_{U}|\left(x^{*},y^{*}\right),D\right)$$

Similar to the mutual information strategy, the second term of Equation (41) can be evaluated using Equation (38). As for the first term $H(Y_{U}|D)$, similar to Equation (38), it can be computed as follows:

(42) $$H\left(Y_{U}|D\right)=\frac{1}{2}\log det\left(R_{Y}\right)+\phi \left(2a,m\right)+\left(a+\frac{m}{2}\right)\left(\Psi \left(a+\frac{m}{2}+1\right))-\Psi \left(a\right)\right)e^{-\frac{{\mu_{Y}}^{T}{R_{Y}}^{-1}\mu_{Y}}{2}+1}$$

where $a=a_{\sigma |D}$, $\mu_{Y}=\mu_{Y|D}$, $\Sigma_{Y}=\Sigma_{Y|D}$, and $R_{Y}=R_{Y|D}$. For $\Delta_{Y}$ it is evaluated as follows:

(43) $$\Delta_{Y}={\mu_{Y}}^{T}{R_{Y}}^{-1}\mu_{Y}$$

where $R_{Y}$ denotes the correlation matrix of the unlabeled samples *Y*, given the training data acquired so far D.

Therefore, substituting from Equations (28) and (42) into Equation (41) results in:

(44) $$\begin{array}{c} I\left(x^{*},Y_{U}\right)=\frac{1}{2}\log det\left(R_{Y|D\cup (x^{*},y^{*})}\right)+\phi \left(2a_{\sigma |D,\cup (x^{*},y^{*})},m\right)+\left(a_{\sigma |D,\cup (x^{*},y^{*})}+\frac{m}{2}\right)\times \\ \left(\Psi \left(a_{\sigma |D,\cup (x^{*},y^{*})}+\frac{m}{2}+1\right))-\Psi \left(a_{\sigma |D,\cup (x^{*},y^{*})}\right)\right)e^{-\frac{\mu_{Y|D,\cup (x^{*},y^{*})}^{T}R_{Y|D,\cup (x^{*},y^{*})}^{-1}\mu_{Y|D,\cup (x^{*},y^{*})}}{2}+1}\\ -\frac{1}{2}\log det\left(R_{Y}\right)+\phi \left(2a,m\right)+\left(a+\frac{m}{2}\right)\left(\Psi \left(a+\frac{m}{2}+1\right))-\Psi \left(a\right)\right)e^{-\frac{{\mu_{Y}}^{T}{R_{Y}}^{-1}\mu_{Y}}{2}+1} \end{array}$$

(45) $$\begin{array}{c} x_{MMI}={arg max}_{x^{*}}\frac{1}{2}\left(\log det\left(R_{Y|D\cup (x^{*},y^{*})}\right)-\log det\left(R_{Y}\right)\right)+\left(a_{\sigma |D,\cup (x^{*},y^{*})}+\frac{m}{2}\right)\times \\ \left(\Psi \left(a_{\sigma |D,\cup (x^{*},y^{*})}+\frac{m}{2}+1\right))-\Psi \left(a_{\sigma |D,\cup (x^{*},y^{*})}\right)\right)e^{-\frac{\mu_{Y|D,\cup (x^{*},y^{*})}^{T}R_{Y|D,\cup (x^{*},y^{*})}^{-1}\mu_{Y|D,\cup (x^{*},y^{*})}}{2}+1}\\ -\left(a+\frac{m}{2}\right)\left(\Psi \left(a+\frac{m}{2}+1\right))-\Psi \left(a\right)\right)e^{-\frac{{\mu_{Y}}^{T}{R_{Y}}^{-1}\mu_{Y}}{2}+1} \end{array}$$

Similar to the previous strategy, we apply three variants of this active learning method using the different active learning schemes: pool-based, query synthesis and query synthesis without pool.

#### 5.2.3. Kullback–Leibler Divergence (KL)

So far, the previously mentioned strategies select the sample revealing the most amount of information for the labels of the other samples. However, this strategy addresses a different aspect. The Kullback–Leibler divergence strategy seeks to acquire samples having the greatest impact on the posterior predictive distribution of the unlabeled samples $p(Y_{U}|X,D)$. So, this method considers the influence of the query sample on the *“distribution”* of the unlabeled samples. To achieve that, this method maximizes the difference in posterior predictive distributions of unlabeled pool $Y_{U}$ before and after querying the query point $\left(x^{*},y^{*}\right)$. The distribution difference is evaluated using the Kullback–Leibler divergence (KL) metric [44]. The Kullback–Leibler divergence metric is an asymmetric distance measure that evaluates the distance between two probability distributions *P* and *Q*. In other words, $D_{KL}\left(P||Q\right)$ measures the information lost when *Q* is used to approximate *P* [44]. The $D_{KL}\left(P||Q\right)$ is defined as follows:

$$D_{KL}\left(P||Q\right)=\int_{-\infty }^{\infty }\;p\left(x\right)\log\frac{p(x)}{q(x)}dx$$

where p(x) and q(x) are the probability density functions to be compared.

It is worth noting that the KL divergence has been employed as a powerful method in Bayesian analysis. For example, Lopez et al. apply the KL divergence to influence analysis [45]. The authors use the KL metric to study the impact of removing one or several observations from data set on the inferences.

In our proposed active learning method, p(x) denotes the posterior predictive distribution of unlabeled example given the query sample $\left(x^{*},y^{*}\right)$, whereby q(x) is the posterior predictive distribution of unlabeled example *prior to* acquiring the query example $\left(x^{*},y^{*}\right)$. The Kullback–Leibler divergence $D_{KL}\left(U|D,x^{*}\right)$ is defined as:

(46) $$D_{KL}\left(U|D,x^{*}\right)=D_{KL}\left(p\left(Y_{U}|D,x^{*},y^{*}\right),p\left(Y_{U}|D\right)\right)$$

We approximate $D_{KL}\left(U|D,x^{*}\right)$ by evaluating the average Kullback–Leibler divergence over all the unlabeled examples of the pool $Y_{U}$.

(47) $$D_{KL}\left(U|D,x^{*}\right)=\frac{1}{\left|U\right|}\sum_{k\in U}D_{KL}\left(p\left(y_{k|D,(x^{*},y^{*})}\right),p\left(y_{k|D}\right)\right)$$

Since the true label $y^{*}$ of the query sample $x^{*}$ is unknown, we use the expectation of $y^{*}$ denoted as $E[y^{*}|\mu_{\beta },x^{*}]$.

As indicated in Section 4, both predictive distributions $p(y_{k|D,(x^{*},y^{*})})$ and $p(y_{k|D})$ follow Student-t distributions. Let $p\left(y_{k|D,(x^{*},y^{*}))}\right)∼t_{2a_{\sigma |D\cup (x^{*},y^{*})}}\left(y_{k},E\left[y_{k}|x_{k},D\cup \left(x^{*},y^{*}\right)\right],{\sigma^{2}}_{y_{k}|x_{k},D\cup \left(x^{*},y^{*}\right)}\right)$. Similarly, the posterior predictive distribution after acquiring $y^{*}$ is denoted as $p\left(y_{k|D}\right)∼t_{2a_{\sigma |D}}\left(y_{k},E\left[y_{k}|x_{k},D\right],{\sigma^{2}}_{y_{k}|x_{k},D}\right)$.

To simplify notation, let $D_{KL}\left(k|D,\left(x^{*},y^{*}\right)\right)$ denote the Kullback–Leibler divergence between the two predictive distributions $D_{KL}\left(p\left(y_{k|D,x^{*},y^{*}}\right),p\left(y_{k|D}\right)\right)$, which is calculated as:

(48) $$D_{KL}\left(k|D,x^{*}\right)=\int_{-\infty }^{\infty }\;p\left(y_{k|D,x^{*},y^{*}}\right)\log\frac{p(y_{k|D,x^{*},y^{*}})}{p(y_{k|D})}dx$$

Substituting with the Student-t distribution formulation Equation (22) into Equation (48):

(49) $$\begin{array}{c} D_{KL}\left(k|D,x^{*}\right)=\int_{-\infty }^{\infty }\;t_{2a_{\sigma |D\cup (x^{*},y^{*})}}\left(y_{k},E\left[y_{k}|x_{k},D\cup \left(x^{*},y^{*}\right)\right],{\sigma^{2}}_{y_{k}|x_{k},D\cup \left(x^{*},y^{*}\right)}\right)\times \\ \log\frac{t_{2a_{\sigma |D\cup (x^{*},y^{*})}}\left(y_{k},E\left[y_{k}|x_{k},D\cup \left(x^{*},y^{*}\right)\right],{\sigma^{2}}_{y_{k}|x_{k},D\cup \left(x^{*},y^{*}\right)}\right)}{t_{2a_{\sigma |D}}\left(y_{k},E\left[y_{k}|x_{k},D\right],{\sigma^{2}}_{y_{k}|x_{k},D}\right)}dx \end{array}$$

where the means and variances of the posterior distributions can be given using the regression equations in Section 4, Equations (23) and (24), respectively.

After substituting from Equation (49) into Equation (47), the Kullback–Leibler divergence $D_{KL}\left(U|D,x^{*}\right)$ is evaluated as:

(50) $$\begin{array}{c} D_{KL}\left(U|D,x^{*}\right)=\frac{1}{\left|U\right|}\sum_{k\in U}\int_{-\infty }^{\infty }\;t_{2a_{\sigma |D\cup (x^{*},y^{*})}}\left(y_{k},E\left[y_{k}|x_{k},D\cup \left(x^{*},y^{*}\right)\right],{\sigma^{2}}_{y_{k}|x_{k},D\cup \left(x^{*},y^{*}\right)}\right)\times \\ \log\frac{t_{2a_{\sigma |D\cup (x^{*},y^{*})}}\left(y_{k},E\left[y_{k}|x_{k},D\cup \left(x^{*},y^{*}\right)\right],{\sigma^{2}}_{y_{k}|x_{k},D\cup \left(x^{*},y^{*}\right)}\right)}{t_{2a_{\sigma |D}}\left(y_{k},E\left[y_{k}|x_{k},D\right],{\sigma^{2}}_{y_{k}|x_{k},D}\right)}dx \end{array}$$

Finally, the query sample $x^{*}$ that maximizes the Kullback–Leibler divergence between the posterior predictive distributions of unlabeled pool $Y_{UL}$ before and after querying the query point $\left(x^{*},y^{*}\right)$ is evaluated as follows:

(51) $$\begin{array}{c} x_{KL}={arg max}_{x^{*}}\frac{1}{\left|U\right|}\sum_{k\in U}\int_{-\infty }^{\infty }\;t_{2a_{\sigma |D\cup (x^{*},y^{*})}}\left(y_{k},E\left[y_{k}|x_{k},D\cup \left(x^{*},y^{*}\right)\right],{\sigma^{2}}_{y_{k}|x_{k},D\cup \left(x^{*},y^{*}\right)}\right)\times \\ \log\frac{t_{2a_{\sigma |D\cup (x^{*},y^{*})}}\left(y_{k},E\left[y_{k}|x_{k},D\cup \left(x^{*},y^{*}\right)\right],{\sigma^{2}}_{y_{k}|x_{k},D\cup \left(x^{*},y^{*}\right)}\right)}{t_{2a_{\sigma |D}}\left(y_{k},E\left[y_{k}|x_{k},D\right],\sigma_{y_{k}|x_{k},D}\right)}dx \end{array}$$

Like the two aforementioned active learning methods, we apply the three variants of active learning settings described in Section 5.1, along with this active learning method.

#### 5.2.4. Model Entropy (ME)

The aforementioned strategies, the two variants of mutual information and Kullback–Leibler divergence, exploit the potential information of the unlabeled pool to guide the query selection process. However, this novel active learning strategy, named model entropy, considers a different aspect. The tmodel entropy method targets the ultimate objective for the exploration, as mentioned in Section 1, which is minimizing the learning model uncertainty. In order to achieve this target, this method emphasizes reducing the learning model uncertainty in terms of the model entropy. Thus, this method queries the data sample that minimizes the model entropy in order to reveal the uncertainty of the underlying model and obtain better estimates of the learning model parameters.

In general, the entropy has been used in several applications such as biological systems [46], financial applications [47] and model selection [48]. However, to the best of our knowledge, the use of the model entropy minimization strategy in the active learning field is novel.

According to Reference [4], the existing work in active learning literature so far mainly addresses the following: minimizing the approximate generalization error [19] and reducing the model uncertainty indirectly either by choosing the example about which the model is most uncertain [15] or by querying the example that produces the maximum model change [12].

For the general regression problem formulation presented in Section 3, using Equation (1), the model entropy of the regression model parameters β can be formulated as follows:

(52) $$H\left(\beta |x^{*},D\right)=-\int_{\beta }p\left(\beta |x^{*},D\right)\log p\left(\beta |x^{*},D\right)\;d\beta$$

Using the Bayesian linear regression formulation presented in Section 4 (see Equation (9)), the model parameter β follows a multivariate Student-t distribution such that:

(53) $$p\left(\beta |D,x^{*},y^{*}\right)∼t_{2a_{\sigma |D\cup (x^{*},y^{*})}}\left(\mu_{\beta |D\cup (x^{*},y^{*})},\frac{b_{\sigma |D\cup (x^{*},y^{*})}}{a_{\sigma |D\cup (x^{*},y^{*})}-1}\Sigma_{\beta |D\cup (x^{*},y^{*})}\right)$$

(54) $$\begin{array}{c} H\left(\beta |x^{*},y^{*}D\right)=-\int_{\beta }t_{2a_{\sigma |D\cup (x^{*},y^{*})}}(\mu_{\beta |D\cup (x^{*},y^{*})},\frac{b_{\sigma |D\cup (x^{*},y^{*})}}{a_{\sigma |D\cup (x^{*},y^{*})}-1}\Sigma_{\beta |D\cup (x^{*},y^{*})})\times \\ \log t_{2a_{\sigma |D\cup (x^{*},y^{*})}}(\mu_{\beta |D\cup (x^{*},y^{*})},\frac{b_{\sigma |D\cup (x^{*},y^{*})}}{a_{\sigma |D\cup (x^{*},y^{*})}-1}\Sigma_{\beta |D\cup (x^{*},y^{*})})\;d\beta \end{array}$$

where $\mu_{\beta |D,(x^{*},y^{*})}$ and $\Sigma_{\beta |D,(x^{*},y^{*})}$ are the posterior mean and covariance matrix of model parameter β, respectively and they can be evaluated using Equations (12) and (13), respectively, according to the Bayesian linear regression formulation described in Section 4. Furthermore, the posterior values, $a_{\sigma |D\cup (x^{*},y^{*})}$ and $b_{\sigma |D\cup (x^{*},y^{*})}$ are evaluated using Equations (7) and (8), respectively.

To simplify notation, let $a^{*}=a_{\sigma |D\cup (x^{*},y^{*})}$, ${\mu_{\beta }}^{*}=\mu_{\beta |D\cup (x^{*},y^{*})}$ and ${\Sigma_{\beta }}^{*}=\frac{b_{\sigma |D\cup (x^{*},y^{*})}}{a_{\sigma |D\cup (x^{*},y^{*})}-1}\Sigma_{\beta |D\cup (x^{*},y^{*})}$. According to Reference [41], the final formulation of the entropy for the multivariate Student-t distribution is given by:

(55) $$H\left(\beta |x^{*},y^{*}D\right)=\frac{1}{2}\log det\left(R_{\beta |D\cup (x^{*},y^{*})}\right)+\phi \left(2a^{*},d\right)+\left(a^{*}+\frac{d}{2}\right)M\left(2a^{*},d,\Delta_{\beta }\right)$$

where $R_{\beta |D\cup (x^{*},y^{*})}$ denotes the correlation matrix of β, *d* is the dimensionality of β, ϕ is a constant depending on *d* and $a^{*}$ and $\Delta_{\beta }={{\mu_{\beta }}^{*}}^{T}{{\Sigma_{\beta }}^{*}}^{-1}{\mu_{\beta }}^{*}$.

(56) $$M\left(2a^{*},d,\Delta_{\beta }\right)=e^{-\frac{{{\mu_{\beta }}^{*}}^{T}{{\Sigma_{\beta }}^{*}}^{-1}{\mu_{\beta }}^{*}}{2}}\sum_{j=0}^{\infty }\frac{1}{j!}\left\{\Psi \left(\frac{d+a^{*}+2j}{2}\right)-\Psi \left(\frac{a^{*}}{2}\right)\right\}$$

Substituting from Equation (36) into Equation (56):

(57) $$M\left(2a^{*},d,\Delta_{\beta }\right)=\left(\left(\Psi \left(a^{*}+\frac{d}{2}+1\right))-\Psi \left(a^{*}\right)\right)\right)e^{-\frac{{{\mu_{\beta }}^{*}}^{T}{{\Sigma_{\beta }}^{*}}^{-1}{\mu_{\beta }}^{*}}{2}+1}$$

Substituting from Equation (56) into Equation (55) results in:

(58) $$\begin{array}{c} H\left(\beta |x^{*},y^{*}D\right)=\frac{1}{2}\log det\left(R_{\beta |D\cup (x^{*},y^{*})}\right)+\phi \left(2a_{D\cup (x^{*},y^{*})},d\right)+\left(a_{D\cup (x^{*},y^{*})}+\frac{d}{2}\right)\times \\ \left(\left(\Psi \left(a_{D\cup (x^{*},y^{*})}+\frac{d}{2}+1\right))-\Psi \left(a_{D\cup (x^{*},y^{*})}\right)\right)\right)e^{-\frac{\left(a_{\sigma |D\cup (x^{*},y^{*})}-1\right){{\mu_{\beta }}^{T}}_{D\cup (x^{*},y^{*})}{{\Sigma_{\beta }}^{-1}}_{D\cup (x^{*},y^{*})}{\mu_{\beta }}_{D\cup (x^{*},y^{*})}}{2b_{\sigma |D\cup (x^{*},y^{*})}}+1} \end{array}$$

However, we could safely ignore the term $\phi (2a^{*},d)$ since it does not depend on the query sample $x^{*}$. Finally, the query sample $x^{*}$ minimizing the model entropy $H(\beta |x^{*},y^{*}D)$ can be estimated as follows:

(59) $$\begin{array}{c} x_{ME}=\underset{x^{*}}{arg min}\frac{1}{2}\log det\left(R_{\beta |D\cup (x^{*},y^{*})}\right)+\left(a_{D\cup (x^{*},y^{*})}+\frac{d}{2}\right)\times \\ \left(\left(\Psi \left(a_{D\cup (x^{*},y^{*})}+\frac{d}{2}+1\right))-\Psi \left(a_{D\cup (x^{*},y^{*})}\right)\right)\right)e^{-\frac{\left(a_{\sigma |D\cup (x^{*},y^{*})}-1\right){{\mu_{\beta }}^{T}}_{D\cup (x^{*},y^{*})}{{\Sigma_{\beta }}^{-1}}_{D\cup (x^{*},y^{*})}{\mu_{\beta }}_{D\cup (x^{*},y^{*})}}{2b_{\sigma |D\cup (x^{*},y^{*})}}+1} \end{array}$$

For this strategy, we apply both of pool-based and query synthesis active learning approaches.

### 5.3. Exploitation-Based Strategies

In this section, we present the exploitation-based active learning strategies for regression that we apply in our proposed framework. Such strategies purely emphasize on maximizing a certain objective function, with no consideration given to the concept of exploration.

First, we describe the greedy strategy that is considered a pure exploitation method. Then, we propose using a novel active learning querying strategies that mainly focus on exploitation but in a less myopic way than the commonly adopted greedy strategy.

#### 5.3.1. Greedy Strategy (G)

This query strategy addresses pure exploitation by querying the sample resulting in the maximum immediate value of the target objective function (reward). We apply this method as a baseline to compare with, where for every iteration, the query sample is chosen to maximize the expected utility function.

Although the greedy strategy is straightforward and simple, it is myopic in since that it purely considers exploitation, which could result in potential revenue loss, since it pays no attention to improving the model predictive power, which could severely affect the resulting decision, which is commonly known as exploration.

(60) $$x_{G}=\underset{x}{arg max}E\left[u\left(x\right)|D\right]$$

where the expected utility function *u* can be expressed as a function of *x* and the regression model coefficients β.

#### 5.3.2. Expected Value of Perfect Information (EVPI)

We propose a decision-theoritic querying approach which is based on the expected value of perfect information (EVPI). Evaluating the expected value of perfect information could be beneficial for active learning since one can evaluate how revealing a certain query sample is valuable. In other words, active learning could be guided to choose data points that do improve the gained expected utility using EVPI. According to Russell and Norvig [49], the expected value of perfect information for revealing a piece of information, named evidence $E_{j}$, given an initial evidence *e* is defined as:

(61) $$E\left[VPI_{e}\left(E_{j}\right)\right]=\sum_{k}P\left(E_{j}=e_{jk}|e\right)E\left[u\left(\alpha_{e_{jk}}|e,E_{j}=e_{jk}\right)\right]-E\left[u\left(\alpha |e\right)\right]$$

where α is the action to be taken and the expected utility of taking action α given the evidence *e* and after revealing $E_{j}$, $E[u\left(\alpha_{e_{jk}}|e,E_{j}=e_{jk}\right)]$ is defined as:

(62) $$E\left[u\left(\alpha_{e_{j}}|e,e_{j}\right)\right]=\max_{a}\;\sum_{s^{\prime }}P\left(Result\left(a\right)=s^{\prime },e,e_{j}\right)u\left(s^{\prime }\right)$$

while the expected utility of taking action α given the evidence *e* and without revealing $E_{j}$ is denoted by E[u(α|e)] and it is defined as:

(63) $$E\left[u\left(\alpha |e\right)\right]=\underset{a}{arg max}\;\sum_{s^{\prime }}P\left(Result\left(a\right)=s^{\prime },e\right)u\left(s^{\prime }\right)$$

We apply the value of information formulation to the active learning with utility maximization. So, the action α is querying a data point $x^{*}$ to obtain its label $y^{*}$. For the initial evidence *e*, it denotes the training labeled data points so far D. Also, $e_{j}$ represents the acquired label $y^{*}$ of the query point $x^{*}$ which represents the piece of information we seek to evaluate.

The expected value of perfect information after querying $x^{*}$ and acquiring its label $y^{*}$ is:

(64) $$E\left[VPI_{e}\left(y^{*}\right)\right]=\int_{y_{j}}P\left(y_{j}|x^{*},D\right)E\left[u\left(x^{*}|D,y_{j}\right)\right]-E\left[u\left(x^{*}|D\right)\right]$$

Accordingly, the expected utility of acquiring the data sample $x^{*}$ given the observed training data so far D and after observing the evidence $y_{j}$, the true value of $y^{*}$, $E[u\left(x^{*}|D,y_{j}\right)]$, can be formulated as:

(65) $$E\left[u\left(x_{y_{j}}^{*}|D,y_{j}\right)\right]=\max_{x}\;E\left[u\left(x|D,y_{j}\right)\right]=E\left[u_{opt}|D,y_{j}\right]$$

where the utility *u* is the target objective function to be maximized, which is conventionally a function of the data point *x* and the model parameters β. ${\mu_{\beta }}^{*}$ denotes the expectation of the updated model parameters β after revealing point *x* and its label value $y_{j}$.

The second term of Equation (64) could be safely ignored, since the objective is to decide whether to acquire the data label $y^{*}$ or not, maximizing EVPI, this term is independent of $y^{*}$. This is implied by the following equation, Equation (66). Consequently, this term does not affect the process of maximizing EVPI.

(66) $$E\left[u\left(x^{*}|D\right)\right]=\max_{x}\;E\left[u\left(x|D\right)\right]=E\left[u_{opt}|D\right]$$

Consequently, the term $E[u_{opt}|D]$ is constant over all query points $x^{*}$, so it could be safely ignored. Then, evaluating the EVPI by substituting from Equation (65) into Equation (64) results in the following formula:

(67) $$E\left[VPI_{D}\left(y^{*}\right)\right]=\int_{y_{j}}P\left(y_{j}|D,x^{*}\right)E\left[u_{opt}|D,y_{j}\right]\;dy_{j}-E\left[u_{opt}|D\right]$$

Finally, maximizing Equation (67) by differentiating it with respect to $x^{*}$, equating the obtained derivative to zero and solving the resulting equation or using any direct optimization method, we get the query point $x^{*}$ of the highest value of the expected value of perfect information as indicated in the following equation.

(68) $$x_{EVPI}=\underset{x^{*}}{arg max}\int_{y_{j}}P\left(y_{j}|D,x^{*}\right)E\left[u_{opt}|D,y_{j}\right]\;dy_{j}-E\left[u_{opt}|D\right]$$

The expected value of perfect information method seems similar to the mean objective cost of uncertainty (MOCU) method proposed in Reference [31] and described in Section 2.5. Both methods can be viewed from decision theory perspective. The MOCU method seeks to minimize the expected regret which is the difference between the gained utility using the current model and the optimal utility. On the other hand, the EVPI method aims to maximize the difference between the optimal utility before and after acquiring a certain evidence. Accordingly, the MOCU method minimizes the deviation from the optimal decision. However, the EVPI method maximizes the expected utility improvement before and after acquiring a certain piece of information.

### 5.4. Balancing Exploration and Exploitation Strategies

This section describes several active learning strategies that seek to achieve the balance between exploration and exploitation.

#### 5.4.1. Upper Confidence Bound (UCB)

The Upper Confidence Bound (UCB) strategy is proposed by Auer et al. in [36] in the context of multi-armed bandit problems [35]. We apply the UCB method as an active learning baseline strategy to compare with. The main advantage of this method is that it combines exploitation and exploration in a simple, yet an elegant way. The UCB strategy picks the unlabeled example maximizing the upper confidence bound of the random variable of interest, representing the utility function *u*.

(69) $$x_{UCB}=\underset{x^{*}}{arg max}\;\;\;E\left[u\left(x^{*}\right)|D\right]+\eta \sigma_{u(x^{*})|D}$$

where *u* is the objective utility function to be maximized, $E[u\left(x^{*}\right)|D]$ and $\sigma_{u(x^{*})|D}$ denote the expected value and the standard deviation of the utility function for query point $x^{*}$ given the training data acquired so far D.

#### 5.4.2. Probabilistic-Based Exploration-Exploitation (PEE)

This active learning strategy is originally inspired by simulated annealing [50]. More specifically, the probabilistic-based exploration-exploitation strategy is built on the ϵ-decreasing greedy algorithm [51]. In order to manage the trade-off between exploration and exploitation, this algorithm combines exploration and exploitation in a probabilistic way. With probability $p_{R}$, the exploration is performed via any exploratory strategy mentioned in Section 5.2 such as mutual information, Kullback–Leibler divergence and model entropy strategies. Furthermore, other exploration strategies in active learning literature can be incorporated into this method, such as uncertain sampling [4,15] and random sampling.

The exploration probability $p_{R}$ is calculated as follows:

(70) $$p_{R}=\alpha^{t-1}$$

where α is less than 1 and *t* is the current time step or iteration number. The exploration probability intuitively decays over time as seen in Equation (70) since the learning model gets to be more robust and capable of performing some exploitation to achieve the ultimate goal of utility maximization.

To implement this strategy, a uniform random variable *Z* is generated, if $Z\leq p_{R}$, any reasonable exploration strategy can be performed, otherwise pure exploitation is applied via maximizing the expected utility (the greedy strategy). However, any other exploitation strategy can be employed.

For the probabilistic-based exploration-exploitation strategy, we have implemented all of our proposed exploration based strategies in Section 5.2 in addition to uncertain sampling and random sampling. To perform exploitation, we use the greedy strategy since it is the simplest method. Although the greedy strategy is myopic since it does not account for enhancing the learning model estimate, in this PEE method the greedy strategy is integrated with an exploration strategy which already achieves an accurate model estimate.

#### 5.4.3. Uncertainty of Strategy (UoS)

Similar to the probabilistic-based exploration-exploitation (PEE) strategy, this proposed active learning method seeks to balance the trade-off between exploration and exploitation in a probabilistic manner. Naturally, active learning querying strategies require a learning model estimate. Furthermore, many active learning strategies including: uncertain sampling [1] and greedy sampling, build their selection decisions entirely based on the learning model estimate. However, active querying methods that fully trust their estimate of the learning model and do not account for the learning model uncertainty could probably yield inaccurate querying decisions. This argument motivates us to design a novel active learning method named Uncertainty of Strategy (UoS). The UoS method accounts for the inherent uncertainty of the querying criterion which is mainly caused by the model uncertainty or due to any other randomness in the active querying method.

The UoS strategy seeks to achieve the balance between exploitation and exploration. The exploitation can be easily performed using the current model estimate, for example, using greedy sampling. On the other hand, the exploration is done as follows: the UoS strategy sets a window of exploration around the active learning strategy’s best estimate of a data point, which is returned by exploitation. The length of the exploration window can be estimated using the model uncertainty as described subsequently.

Let the query sample $x_{UoS}$ follow a Gaussian distribution as follows:

(71) $$x_{UoS}∼N\left(x_{s},{\sigma_{s}}^{2}\right)$$

where the mean of this Gaussian distribution $x_{s}$ represents the data point returned using pure exploitation. For the Gaussian distribution’s variance ${\sigma_{s}}^{2}$, it essentially depends on the model uncertainty. We estimate the strategy variance ${\sigma_{s}}^{2}$ using two different ways. The first method, named UoS-1 assumes that the strategy variance ${\sigma_{s}}^{2}$ is proportional to the model uncertainty, where the model uncertainty is estimated using the covariance matrix of the vector of model parameters β. Equation (72) defines the estimation of ${\sigma_{s}}^{2}$ in terms of the model uncertainty.

(72) $${\sigma_{s}}^{2}=K\times \frac{b_{\sigma |D}}{a_{\sigma |D}-1}trace\left[\Sigma_{\beta |D}\right]$$

where *K* is a parameter set to adapt the units of the query point and the model parameters and to control the exploration/exploitation trade-off.

Like the PEE method, we set the *K* parameter to be time variant, in order to shrink the exploration window as iterations proceed since the model would become more reliable, so more emphasis should be devoted to exploitation.

(73) $$K=Z^{t-1}$$

The second method, named UoS-2, estimates the strategy variance ${\sigma_{s}}^{2}$ empirically using a simple Monte Carlo simulation. This simulation runs for *n* iterations, where each iteration *i* proceeds as follows: first, an instance of model parameters vector $\beta_{i}$ is generated according to the multivariate Student-T distribution using Equation (9). Then, this model parameters’ instance $\beta_{i}$ is used to evaluate the query point using a pure exploitation strategy ${x_{s}}_{i}$, this is, generally, a simple step as done in greedy strategy Equation (60) for example. Finally, after the *n* iterations finish, the strategy variance ${\sigma_{s}}^{2}$ is statistically evaluated as follows:

(74) $${\sigma_{s}}^{2}=\frac{K}{n-1}\sum_{i=1}^{n}{\left({x_{s}}_{i}-{\bar{x}}_{s}\right)}^{2}$$

where *K* is a parameter for adapting units of the query point and model variance and for controlling the exploration-exploitation trade-off, akin to the UoS-1 method, *K* is defined in Equation (73). The expectation of strategy returned points $\bar{x_{s}}$ is evaluated as the statistical mean over the *n* iterations as follows:

(75) $${\bar{x}}_{s}=\frac{1}{n}\sum_{i=1}^{n}{x_{s}}_{i}$$

The UoS-2 method is akin to the UoS-1 method for evaluating the strategy variance ${\sigma_{s}}^{2}$ defined in Equation (72) in since that it depends on the model uncertainty. However, this dependency is incorporated indirectly through the described Monte Carlo simulation. Algorithm 4 describes the UoS-2 method.

The proposed UoS active learning method, with its two variants, is general and can be combined with any exploitation-based strategy. Furthermore, the UoS method could be integrated with other popular active learning methods such as uncertain sampling [1] and expected model change [12], since most active learning strategies adhere to the greedy approach by querying a data point that maximizes or minimizes a certain selection criterion. In other words, the UoS querying approach could be used as a wrapper for any ordinary active learning method *S* that is greedy in its nature or does not consider the uncertainty of the learned model. This could be achieved by using *S* as the exploitation-based strategy used in the UoS method and adopting either of the two variants of the UoS method to estimate the strategy uncertainty.

**Algorithm 4** The Uncertainty of Sampling Second Variant (UoS-2) Querying Method
**Input:** A dataset $D=(x_{i},y_{i})$, an exploitation active learning strategy *S*, the number of simulation iterations *n* and a scaling parameter *K*.**Output:** A query sample $x^{*}$.Train the regression model using the training samples D to obtain the mean $\mu_{\beta |D}$ and the covariance $\Sigma_{\beta |D}$ of the model parameters’ vector β and the posterior estimates of $a_{\sigma |D}$ and $b_{\sigma |D}$.**for**i=1 to *n* **do**Sample $\beta_{i}$ from $t_{2a_{\sigma |D}}\left(\mu_{\beta |D},\frac{b_{\sigma |D}}{a_{\sigma |D}}\Sigma_{\beta |D}\right)$.${x_{s}}_{i}\leftarrow$ the query sample returned after applying exploitation strategy *S*, using the sampled model parameters $\beta_{i}$.
**end for**
Evaluate the average query sample ${\bar{x}}_{s}$: ${\bar{x}}_{s}=\frac{1}{n}\sum_{i=1}^{n}{x_{s}}_{i}$${\sigma_{s}}^{2}\leftarrow \frac{K}{n-1}\sum_{i=1}^{n}{\left({x_{s}}_{i}-{\bar{x}}_{s}\right)}^{2}$.$x^{*}\leftarrow$ Generate a query sample according to a Gaussian distribution as follows: $N({\bar{x}}_{s},{\sigma_{s}}^{2})$.


#### 5.4.4. Utility minus Model Entropy (UME)

The Utility minus Model Entropy (UME) strategy controls the trade-off between exploration and exploitation in a novel way. The UME querying method adjusts the exploration and exploitation by explicitly modeling both of them in a formulated single objective function. Specifically, the UME method combines the ultimate goal of maximizing a certain utility function *u*, representing exploitation and the secondary but necessary target of minimizing model entropy, representing exploration, into one objective function. Then, the strategy queries the data sample $x^{*}$ maximizing this hybrid objective as follows:

(76) $$x_{UME}=\underset{x^{*}}{arg max}E\left[u\left(x^{*}\right)|D\right]-\eta H\left[\beta |x^{*},D\right]$$

where the model entropy $H[\beta |p^{*},D]$ is evaluated using Equation (58) and η is the exploration- exploitation trade-off control parameter. We conveniently let η be exponentially decreasing in time according to Equation (77). At early iterations, more emphasis is imposed on exploration to have better estimate for model parameters, however at later iterations since the model estimates get more robust over time, then more attention should be paid to the exploitation.

(77) $$\eta =\eta_{0}e^{-\alpha t}$$

where *t* is iteration number and α>0.

Substituting from Equation (58) into Equation (76) results in:

(78) $$\begin{array}{c} x_{UME}={arg max}_{x^{*}}E\left[u\left(x^{*}\right)|D\right]-\frac{\eta }{2}\log det\left(R_{\beta |D\cup (x^{*},y^{*})}\right)+\phi \left(2a_{D\cup (x^{*},y^{*})},d\right)+\\ \left(a_{D\cup (x^{*},y^{*})}+\frac{d}{2}\right)\left(\left(\Psi \left(a_{D\cup (x^{*},y^{*})}+\frac{d}{2}+1\right))-\Psi \left(a_{D\cup (x^{*},y^{*})}\right)\right)\right)\\ e^{-\frac{\left(a_{\sigma |D\cup (x^{*},y^{*})}-1\right){{\mu_{\beta }}^{T}}_{D\cup (x^{*},y^{*})}{{\Sigma_{\beta }}^{-1}}_{D\cup (x^{*},y^{*})}{\mu_{\beta }}_{D\cup (x^{*},y^{*})}}{2b_{\sigma |D\cup (x^{*},y^{*})}}+1} \end{array}$$

Since $\phi (2a^{*},d)$ does not depend on the query sample $x^{*}$, then:

(79) $$\begin{array}{c} x_{UME}={arg max}_{x^{*}}E\left[u\left(x^{*}\right)|D\right]-\frac{\eta }{2}\log det\left(R_{\beta |D\cup (x^{*},y^{*})}\right)\left(a_{D\cup (x^{*},y^{*})}+\frac{d}{2}\right)\times \\ \left(\left(\Psi \left(a_{D\cup (x^{*},y^{*})}+\frac{d}{2}+1\right))-\Psi \left(a_{D\cup (x^{*},y^{*})}\right)\right)\right)e^{-\frac{\left(a_{\sigma |D\cup (x^{*},y^{*})}-1\right){{\mu_{\beta }}^{T}}_{D\cup (x^{*},y^{*})}{{\Sigma_{\beta }}^{-1}}_{D\cup (x^{*},y^{*})}{\mu_{\beta }}_{D\cup (x^{*},y^{*})}}{2b_{\sigma |D\cup (x^{*},y^{*})}}+1} \end{array}$$

## 6. Case Study: Dynamic Pricing with Demand Learning

We apply the proposed active learning framework described in Section 5 to a real-world application which is dynamic pricing for revenue maximization in case of unknown behavior of the customers’ demand.

The main challenge of dynamic pricing with unknown demand is that the chosen prices should achieve some balance between exploitation and exploration. Exploitation represents choosing prices aiming to maximize the achieved revenue. On the other hand, exploration selects prices that promote learning the demand model parameters. This motivates us to apply our proposed active learning framework in Figure 1 to this application.

We assume a linear demand elasticity for modeling the customers’ demand behavior as typically used in the economics/finance literature (see Equation (80)). The price is the main controlling variable for demand. We assume a monopolist seller, who has a sufficient inventory to satisfy all potential demand and we, specifically, consider pricing a single product over a finite selling horizon *T*.

The linear demand model equation is defined as follows:

(80) y=a+bp+ϵ

such that b<0 and $\epsilon ∼N(0,\sigma^{2})$.

The parameter *b* represents the price-demand sensitivity, so it is naturally negative since the price and demand have an inverse relationship. For example, if price rises by 10%, demand would diminish, On the other hand, when price decreases by 10%, demand would increase.

In order to estimate the demand model parameters *a* and *b* defined in Equation (80), we apply the Bayesian linear regression model described in Section 4. We employ the active learning framework with its different query generation schemes defined in Section 5.1 and described in detail in Algorithm 1, Algorithm 2 and Algorithm 3. Applying active learning formulation to the dynamic pricing problem, the training data D consists of some pairs of prices and their corresponding demands $\left(p_{i},y_{i}\right)$. In addition, the query point $x^{*}$ denotes the vector $\left[1\;\;p^{*}\right]$. For this application, the utility function after querying a certain price *p* represents the gained revenue *R*, which is defined as follows:

(81) R=p(a+bp)

where *a* and *b* are the demand model parameters defined in Equation (80).

### 6.1. Active Learning Framework Application

In this section, we apply the active learning formulations represented in Section 5 to the dynamic pricing with demand learning problem. First, the exploration-based strategies hinge on minimizing the regression model error, without considering the utility function *u* in their formulations. So, the formulations presented in Section 5.2 can be exactly used for the underlying dynamic pricing application. On the other hand, for the exploitation-based and the balancing strategies described in Section 5.3 and Section 5.4 respectively, specific formulations should be derived for the considered application, setting the utility function *u* to the gained revenue defined in Equation (81).

#### 6.1.1. Exploration-Based Strategies

In our experiments, we apply the four presented strategies in Section 5.2 with pool-based and query synthesis schemes. Moreover, for mutual information, modified mutual information and Kullback–Leibler divergence, we implement the query synthesis approach without a predefined pool as described in Section 5.1 and Algorithm 3. When applying the query synthesis method without a predefined pool to the dynamic pricing problem, we construct *U* defined in Algorithm 3 as follows: since the dynamic pricing application has one controlling variable, the product price, we consider the range of all potential prices between $p_{min}$ and $p_{max}$ and along the active learning iterations, we exclude the prices that are previously queried, added to the training set $D_{L}$. This set of prices *P* are used as unlabeled samples for evaluating the information-theoretic metrics as defined in (Equations (25), (41) and (47)).

#### 6.1.2. Exploitation-Based Strategies

In this section, we apply the exploitation-based strategies introduced in Section 5.3 to the dynamic pricing with demand learning problem.

- Greedy Strategy
  Given Equation (60) and setting the utility function *u*, to the gained revenue defined in Equation (81) results in:
  (82) $$p_{G}=\underset{p^{*}}{arg max}\;\;\;E\left[R|p^{*},D\right]$$
  Using the revenue definition in Equation (81), the expected revenue $E[R|p^{*},D]$ for any price $p^{*}$ is evaluated using:
  (83) $$E\left[R^{*}|p,D\right]=p^{*}\left({x^{*}}^{T}\mu_{\beta |D}\right)$$
  where $x^{*}=\left[1\;\;p^{*}\right]$.
  We apply the greedy strategy in pool-based setting. In addition, we apply it in the query synthesis setting as well by maximizing the expected revenue as stated in Equation (82), using any optimization method or even using a simple grid search if the range of prices between $p_{min}$ and $p_{max}$ is limited.
  By differentiating the expected revenue $E[R|p^{*}]$ w.r.t price $p^{*}$, the myopic price $p_{G}$ maximizing the expected immediate revenue would be calculated as follows:
  (84) $$p_{G}=\frac{-\hat{a}|D}{2\hat{b}|D}$$
  where $\hat{a}|D$ and $\hat{b}|D$ are the estimates of the demand model parameters *a* and *b* defined in Equation (80) using the labeled data gathered so far D.
- Expected Value of Perfect Information (EVPI)
  When applying the value of information strategy to the considered problem, the action α defined in Equation (61) is querying a price $p^{*}$. The initial evidence *e* represents the training labeled data points so far D. Similarly, $e_{j}$ denotes the acquired demand *y* of the query price $p^{*}$, which represents the piece of information we seek to evaluate.
  Accordingly, for the considered problem, the expected utility of taking action $p^{*}$ given the evidence D and after revealing evidence $y_{j}$, the expected utility term, $EU(\alpha_{e_{j}k}|e,E_{j}=e_{jk})$, defined in Equation (65) can be formulated as:
  (85) $$E\left[u\left(p_{y_{j}}^{*}|D,y_{j}\right)\right]=\max_{p}\;R\left(p|D,y_{j}\right)$$
  where the utility *u* can be set to the immediate revenue *R*.
  Using the linear demand model defined in Equation (80) and then applying the optimal price maximizing the immediate revenue defined in Equation (84) of the greedy strategy. Consequently, $E[u\left(p_{y_{j}}^{*}|D,y_{j}\right)]$ would be defined as follows:
  (86) $$E\left[u\left(p_{y_{j}}^{*}|D,y_{j}\right)\right]=\frac{-{\hat{a}}^{2}|D,\left(x^{*},y_{j}\right)}{4\hat{b}|D,\left(x^{*},y_{j}\right)}$$
  The second term of Equation (61) could be safely ignored, since the objective is to experiment a price $p^{*}$, maximizing EVPI and this term is independent of $p^{*}$. This is implied by the following equation, Equation (87), it can be observed that this term does not affect the process of maximizing EVPI.
  (87) $$EU\left(p^{*}|D\right)=\max_{p}\;R\left(p|D,p^{*}\right)=\max_{p}\;R\left(p|D\right)=\frac{-{\hat{a}}^{2}|D}{4\hat{b}|D}$$
  Then, evaluating the EVPI method for revenue maximization problem, by substituting from Equation (86) into Equation (61) results in the following formula:
  (88) $$EVPI_{D}\left(y^{*}\right)=\int_{y_{j}}P\left(y_{j}|D,x^{*}\right)\frac{-{\hat{a}}^{2}|D,\left(x^{*},y_{j}\right)}{4\hat{b}|D,\left(x^{*},y_{j}\right)}\;dy_{j}$$
  Finally, maximizing Equation (88) by differentiating it with respect to $p^{*}$, equating the derivative to zero and solving the resulting equation or using any direct optimization method, we get the price maximizing the expected value of perfect information as follows:
  (89) $$p_{EVPI}=\overset{*}{\underset{x}{arg max}}\int_{y_{j}}P\left(y_{j}|D,x^{*}\right)\frac{-{\hat{a}}^{2}|D,\left(x^{*},y_{j}\right)}{4\hat{b}|D,\left(x^{*},y_{j}\right)}\;dy_{j}$$

#### 6.1.3. Balancing Exploration and Exploitation Strategies

In this section, we consider applying the balancing strategies that combine both aspects of exploration and exploitation and attempt to achieve balance between both of them.

- Upper Confidence Bound (UCB)
  Applying the UCB strategy to the dynamic pricing problem and setting the utility function *u* defined in Equation (69) to the immediate revenue *R* results in:
  (90) $$p_{UCB}=\underset{p^{*}}{arg max}\;\;\;E\left[R|p^{*},D\right]+\eta \sigma_{R|p^{*},D}$$
  where $E[R|p^{*},D]$ and $\sigma_{R|p^{*},D}$ are the expectation and the standard deviation of the estimated immediate gained revenue *R* in response to price $p^{*}$ and using training data labeled so far D.
  The expected revenue $E[R^{*}|x^{*},D]$ is calculated as:
  (91) $$E\left[R^{*}|p^{*},D\right]=p^{*}E\left[y|x^{*},D\right]$$
  where the expected demand $E[y|x^{*},D]$ is computed using the Bayesian linear regression (see Equation (23)) presented in Section 4.
  Accordingly:
  (92) $$E\left[R^{*}|x^{*},D\right]=p^{*}\left({x^{*}}^{T}\mu_{\beta }|D\right)$$
  Using revenue definition in Equation (81) and the posterior variance for demand defined in Equation (24), the variance of revenue $\sigma_{R^{*}|x^{*},D}^{2}$ is calculated as follows:
  (93) $$\sigma_{R^{*}|x^{*},D}^{2}={p^{*}}^{2}\frac{b_{\sigma |D}}{a_{\sigma |D}-1}\left(1+{x^{*}}^{T}\Sigma_{\beta |D}x^{*}\right)$$
  Substituting from Equations (92) and (93) into Equation (90), then the price maximizing the UCB criterion can be evaluated as defined in Equation (94).
  (94) $$p_{UCB}=\underset{p^{*}}{arg max}\;\;\;p^{*}\left({x^{*}}^{T}\mu_{\beta |D}\right)+\eta p^{*}\sqrt{\frac{b_{\sigma |D}}{a_{\sigma |D}-1}\left(1+{x^{*}}^{T}\Sigma_{\beta |D}x^{*}\right)}$$
  where $\Sigma_{\beta |D}$ is evaluated using Equation (6). For the Gamma distribution parameters $a_{\sigma |D}$ and $b_{\sigma |D}$, they are evaluated using Equations (7) and (8), respectively.
- Probabilistic-based Exploration-Exploitation (PEE)
  In our experiments, we apply several instances of this hybrid strategy. We combine the pure exploitation, greedy, strategy as defined in Equation (84), with all the proposed exploration-based methods in addition to the popular active learning method, uncertain sampling [15] and we apply random sampling as a representative for random exploration.
- Uncertainty of Strategy (UoS)
  For the uncertainty of strategy method, defined in Equation (71), the resulting price $p_{UoS}$ follows a Gaussian distribution:
  (95) $$p_{UoS}∼N\left(p_{s},{\sigma_{s}}^{2}\right)$$
  where the mean of this Gaussian distribution, $p_{s}$, represents the price returned using pure exploitation, which is the greedy strategy as defined in Equation (84). Regarding the variance of strategy ${\sigma_{s}}^{2}$, it can be evaluated using two variants: in terms of model uncertainty and using Monte Carlo simulation as described in Section 5.4, specifically using Equations (72) and (74), respectively.
- Utility minus Model Entropy (UME)
  The UME criterion as defined in Equation (76) is a function of the utility which is the immediate revenue and the model entropy. Consequently, substituting from Equations (31), (58) and (92) into Equation (76) results in the following equation:
  (96) $$\begin{array}{c} p_{UME}=\underset{p^{*}}{arg max}p^{*}\left({x^{*}}^{T}\mu_{\beta }|D\right)-\frac{\eta }{2}\log det\left(R_{\beta |D\cup (x^{*},y^{*})}\right)\left(a_{D\cup (x^{*},y^{*})}+\frac{d}{2}\right)\times \\ \left(\left(\Psi \left(a_{D\cup (x^{*},y^{*})}+\frac{d}{2}+1\right))-\Psi \left(a_{D\cup (x^{*},y^{*})}\right)\right)\right)e^{-\frac{\left(a_{\sigma |D\cup (x^{*},y^{*})}-1\right){{\mu_{\beta }}^{T}}_{D\cup (x^{*},y^{*})}{{\Sigma_{\beta }}^{-1}}_{D\cup (x^{*},y^{*})}{\mu_{\beta }}_{D\cup (x^{*},y^{*})}}{2b_{\sigma |D\cup (x^{*},y^{*})}}+1} \end{array}$$
  where $x^{*}=\left[1\;\;\;p^{*}\right]$ and *d* is the dimensionality which equals to 2 in the dynamic pricing application, with linear demand elasticity as defined in Equation (80). Therefore, by differentiating the objective function defined in Equation (96) and equating the resulting equation to zero, we can get the price maximizing the UME.

## 7. Experiments

### 7.1. Experimental Setup

In order to evaluate the performance of the proposed active learning framework summarized in Figure 1, as well as three baseline active learning methods including: random sampling (RS), the greedy or myopic strategy (check Section 5.3.1) and upper confidence bound method, which is intensively used in the multi armed bandit context [36] (see Section 5.4.1). In our experiments, we apply the proposed active learning framework to the dynamic pricing with demand learning problem described in Section 6. In the presented experiments, we mainly focus on analyzing the exploitation strategies introduced in Section 5.3 and the strategies balancing between exploitation and exploration presented in Section 5.4 since the main interest of the paper is applying active learning to utility optimization, which is exploitation.

In this work, we aim to perform a qualitative analysis to evaluate the performance of the pool-based approach versus the query synthesis approach since the query synthesis approach is computationally more efficient than the commonly adopted pool-based approach. Furthermore, the query synthesis approach could be more beneficial for objective optimization, such as maximizing revenue or even minimizing the learning model error, since it is not restricted to a certain given pool of samples. For mostly, all of the proposed active learning strategies including: our proposed strategies and the baseline methods, we implement two variants: one in pool-based setting and the other using query synthesis. In addition to these two active learning schemes, we further apply the third method, query synthesis without a predefined pool described in Section 5.1, to our proposed active learning methods that require the existence of a pool of unlabeled samples such as mutual information (MI), modified mutual Information (MMI) and Kullback–Leibler divergence (KL), in addition to the probabilistic-based exploration-exploitation (PEE) methods using either of MI, MMI or KL strategies for exploration.

In our experiments, we experiment different variants of the the probabilistic-based exploration- exploitation (PEE) approach combining greedy exploitation strategy with several exploration methods including our proposed approaches described in Section 5.2 in addition to random sampling and uncertain sampling [15]. The implemented PEE methods combining our proposed exploration methods presented in Section 5.2 with greedy sampling are denoted as KL-G, MI-G, MMMI-G and ME-G. In addition, we denote combining random and greedy sampling as (RS-G). Similarly, the method combining uncertain and greedy sampling is denoted as (US-G).

Some strategies such as the two variants of uncertainty of strategy (UoS) are basically designed so that the query point is generated or derived by optimizing an objective function. So, for these strategies we consider the query synthesis approach only since the pool-based approach does not apply for UoS.

In the adopted experiments, we apply the Bayesian linear regression model with conjugate prior of the model parameters β and σ, as described in Section 4, for estimating the demand at each iteration.

We conduct our experiments on synthetic and real datasets. The advantage of using artificial data is that the true model parameters β=[a;b] are known. Accordingly, the ground truth value for the objective function, that is, the gained revenue, can be accurately computed with the knowledge of the true optimal model parameters as defined in Equation (81). Moreover, the estimation error of the model parameters β can be properly evaluated.

#### Evaluation Metrics

We assess the performance of our proposed active learning framework in terms of two aspects: the gained utility (revenue) and the accuracy of estimating the regression model parameters.

In order to evaluate the utility maximization, we measure the revenue gain or a normalized version of the total discounted utility $u_{T}$ achieved in the considered time period as defined in Equation (97). We adopt discounted utilities to place more emphasis on getting rewards soon, as widely used in reinforecement learning [52].

(97) $$u_{Gain}=\frac{\sum_{i=1}^{T}\gamma^{i-1}u_{i}}{\sum_{i=1}^{T}\gamma^{i-1}u_{opt}}$$

where $u_{i}$ is the revenue obtained at iteration *i* and $u_{opt}$ is the optimal revenue given the true model parameters *a* and *b*, which is calculated as:

(98) $$u_{opt}=p_{opt}\left(a+bp_{opt}\right)$$

where $p_{opt}$ is the optimal price, which equals to $\frac{-a}{2b}$ in case of linear demand model defined in Equation (80). Simplifying the term $\sum_{i=1}^{T}\gamma^{i-1}$ using the summation of geometric series formula, as follows:

(99) $$u_{Gain}=\frac{\sum_{i=1}^{T}\gamma^{i-1}u_{i}}{\left(1-\gamma^{T}\right)/\left(1-\gamma \right)u_{opt}}$$

For the applications where the optimal utility is not known or cannot be evaluated, the total discounted utility can be used as an evaluation metric.

Concerning the demand model estimation error, we evaluate it in terms of the deviation of the final estimated demand model parameters $\Delta_{\beta }$, from the true parameters β as indicated in Equation (100). The final model estimate is evaluated using the expectation of β given the training data $\mu_{\beta |D}$, as defined in Section 4.

(100) $$\Delta_{\beta }=\frac{||\beta -\mu_{\beta |D}{\left|\right|}_{2}}{{\left||\beta |\right|}_{2}}$$

### 7.2. Experiments Using Synthetic Datasets

We perform a Monte Carlo simulation, generating 12 synthetic datasets of different parameters *a*, *b* and noise levels σ. We use two values for *a*, a=100 and a=1000. For each value for *a*, we adopt three different values for the sensitivity parameter *b* representing elastic demand (b=−2), neutral demand (b=−1) and inelastic demand (b=−0.5). Two different values are adopted for the noise parameter σ representing low (5%) and high (40%) noise levels. Investigating different noise levels enables us to analyze the impact of the noise on the different active learning strategies and evaluate their immunity towards noise. Moreover, for the dynamic pricing problem, we use different noise levels as a way for aggregating all other influencing factors that could affect the demand and may be hard to model, such as competition, seasonality or perishability of the products.

For each dataset, we run the experiment 10 runs and we present the average results over the runs. The synthetic datasets are created as follows: first, we generate N=1000 price points from a Gaussian distribution with mean $\mu_{p}$ and variance ${\sigma_{p}}^{2}$. Then, we assign values for demand elasticity parameters *a* and *b*. After that, assuming a linear demand model, we calculate the corresponding demands using Equation (80). We express the noise level parameter σ in terms of a percentage of the maximum possible demand *a*.

In our experiments, we set $\mu_{p}$ and $\sigma_{p}$ of the Gaussian distribution used for generating the pricing data, using the pricing boundaries given by the seller $p_{min}$ and $p_{max}$. For $\mu_{p}$, it is the mean price of the prices in range of the $\left[p_{min,}p_{max}\right]$, which equals to $\frac{p_{min}+p_{max}}{2}$. Similarly, for the standard deviation $sigma_{p}$, it is estimated using the standard deviation of the potential prices in the range of $\left[p_{min},p_{max}\right]$. We set multivariate Gaussian prior for β as follows: μ=[10,−0.5], $\Sigma =10^{4}I$. For the inverse Gamma prior distribution parameters of the noise parameter $\sigma^{2}$, we set $a_{\sigma }=2$ and $b_{\sigma }=1$.

The simulation proceeds as follows: for each problem, we generate a pool of price-demand data points, starting with a very limited number of data points, $N_{init}=3$ points, then we train a Bayesian regression model to obtain an initial estimate for the model parameters β. After that, we run the different exploitation and balancing active learning strategies described in Section 5.3 and Section 5.4, respectively, with different schemes: pool-based, query synthesis and query synthesis without a predefined pool. For the query synthesis strategies (with and without pool variants), we assume that there is an oracle revealing the true demand value $y^{*}$ for the chosen query point $x^{*}$. For each active learning strategy, we evaluate its performance by measuring the percentage revenue gain defined in Equation (99) and model estimation error defined in Equation (100).

Generally, most of the strategies balancing between exploration and exploitation have a hyper-parameter that controls the trade-off between exploration and exploitation. We set the controlling parameters of the balancing strategies introduced in Section 5.4 as follows: for the UCB method, the η parameter in Equation (69) is set to 0.01. For the PEE method, the α parameter in Equation (70) is set to 0.7. The *K* parameter of UoS strategy first variant, UoS-1, the parameter *Z* in Equation (73) is set to 0.5, while it is set to 0.7 for the second variant UoS-2. Finally, we set α parameter defined in Equation (77), such that at the last iteration *T*, where the exploration is nearly diminished, η equals to a small value: η=0.3. Regarding the $\eta_{0}$ parameter of the same equation, Equation (77), we use values to let the impacts of the exploitation and exploration be comparable at the first iteration.

For the price-demand curve estimation problem, we enforce a constraint that the chosen price $p^{*}$ at each iteration is within the pricing interval defined by the seller where the minimum allowable price is $p_{min}$ and the maximum possible price $p_{max}$, accordingly $p_{min}\leq p^{*}\leq p_{max}$. The active learning loop continues till reaching a certain predefined number of iterations T=100. For the pool-based strategies, the pool size N=1000. We set the discount factor of revenue gained γ used in Equation (99) to 0.99.

The average results for the revenue gain and the regression model estimation error for different active learning strategies using different noise levels are represented in Table 1 and Table 2, respectively.

One of the main contributions of this work is to perform a comparative analysis between the query synthesis and pool-based active learning approaches and demonstrate the benefits of applying active learning query synthesis based strategies for utility maximization. Accordingly, in the performed experiments, for each active learning, except random sampling since it is considered a passive learning method, we adopt a pool-based version and a query synthesis one. We provide an empirical analysis between both approaches. We include both aspects of the achieved revenue gain and model estimation error. Table 3 and Table 4 represent the average revenue gain and the average model estimation error, respectively, for pool-based methods versus query synthesis ones.

In addition, in order to investigate the superiority of either active learning approach, we evaluate the percentage of the pool-based strategies versus the query synthesis strategies existing in the top-10 performing methods in terms of achieving revenue gain, averaged over the different synthetic datasets, as presented in Table 5. Similarly, Table 6 shows the percentage of strategies from both approaches, pool-based and query synthesis, placed in the top-10 strategies achieving minimum regression model estimation error.

### 7.3. Experiments Using Real Datasets

To have the parameters more realistic, we have used several real datasets described in Table 7. We have gathered the first dataset in the table, transport, online though surveying. The dataset is a transportation ticket pricing data, where we ask users about the minimum and maximum fares they would pay for an economy class bus ticket of an air-conditioned bus between any general two cities, City A and City B, such that City A is away around 220 km from City B. We collected 41 responses from different users. In order to have data in the form of price and demand pairs, we perform the following. For each price, we calculate the corresponding demand as the number of users who can afford this price according to the minimum and maximum prices of the data.

For beef dataset, it is obtained from the USDA Red Meats Yearbook [53]. The sugar dataset is adopted from Reference [54] and the spirits dataset is originated from Reference [55]. Finally, the coke dataset is adopted from Reference [56].

There is one hurdle in using such real datasets. In our proposed active learning framework, especially the query synthesis approach, the chosen data point or chosen price $p^{*}$ could potentially be outside the available prices provided in the dataset. Thus, we utilize the dataset mainly for estimating linear demand model parameters vector β using ordinary least squares linear regression. Concerning the noise parameter $\sigma^{2}$, we estimate it using the maximum likelihood estimator. The estimated model parameters $\hat{a}$, $\hat{b}$ and $\hat{\sigma }$ for all the real datasets, are listed in Table 7. Using the obtained demand model parameters, we generate synthetic data using these parameters, with the same methodology described in Section 7.2. However, the mean and variance of the Gaussian distribution for generating pricing data $mu_{p}$ and $v_{p}$, are estimated using the original prices of the real datasets.

Table 8 and Table 9 represent the revenue gain and the estimation error of the regression model parameters, respectively, for the five considered real datasets described in Table 7.

Table 10 summarizes the average utility (revenue) gain and average model percentage error, averaged over the five real datasets described in Table 7, for all the considered active learning strategies.

We have experimented different values for the number of initial training points $N_{init}$ in order to evaluate the impact of varying the number of initial training points on the performance of the different active learning methods. Table 11 and Table 12 show the average revenue gain and model percentage error, respectively, averaged over the five considered real datasets described in Table 7. For space considerations, we include the results of this experiment for the real datasets only. The synthetic datasets exhibit a very similar behavior.

Similar to the synthetic datasets, we compare both active learning approaches, pool-based and query synthesis over the five real datasets in terms of the revenue gain and the model estimation error. Thus, Table 13 and Table 14 demonstrate the average revenue gain and average model estimation error for both approaches over the five real datasets presented in Table 7.

The percentage of pool-based strategies versus query synthesis strategies ranked within the top-10 strategies in terms of the revenue gain is presented in Table 15. Similarly, for model estimation error, Table 16 shows the percentage of strategies of both active learning approaches placed within the top-10 strategies achieving the least model estimation error.

## 8. Discussion

In this section, we investigate the empirical results presented in Section 7. The main findings inferred from the experimental results are summarized as follows:

- It is evident from the presented results presented in Table 1 and Table 10, that our proposed active learning strategies, especially the balancing methods, outperform the standard baselines: the upper confidence bound method, greedy sampling and random sampling, in terms of the achieved utility function (the revenue gain). There are several reasons for this compelling performance.
  First, our proposed balancing methods attain the balance between exploration and exploitation using several novel approaches as described in Section 5.4. For example, for the proposed uncertainty of sampling method (UoS), it combines both aspects of utility maximization and regression model estimation in a probabilistic way, where the exploration is controlled using the model uncertainty.
  In addition, the utility minus entropy (UME) method incorporates the model uncertainty, in addition to the utility function into one hybrid objective function to be optimized, as indicated in Equation (76). The explicit formulation of exploration in the active learning selection criterion imposes an emphasis over the exploration in order to obtain accurate model estimation and hence achieve high future utility returns along the active learning iterations.
  Finally, in the probabilistic-based exploration-exploitation method, we employ several powerful exploration methods, with the pure exploration method, the simple greedy sampling. The proposed exploration methods presented in Section 5.2, which are Kullback–Leibler divergence, mutual information and model entropy, have a great impact on estimating the regression model parameters, which indirectly helps boosting the gained utility (revenue).
- Table 1 shows the revenue gain for different artificial datasets, using different noise levels. It could be observed from this table that our proposed balancing strategies between exploration and exploitation such as the two variants of UoS, the four variants of PEE method combining the information theoretic exploration and pure exploitation: KL-G, MI-G and MMMI-G, ME-G and UME, show a significant revenue gain compared to the pure exploitation strategies such as greedy sampling and EVPI, especially for noisy datasets where σ=40%.
- Moreover, Table 1 indicates that the proposed balancing strategies outperform the baselines including: random sampling, greedy method and the UCB method. It can be observed that our proposed balancing strategies yield a substantial utility (revenue) gain in case of large noise 40%. For example, the KL-G strategy in both pool-based and synthetic settings, achieves around 2%–4% improvement, on average, over greedy sampling (GS). Furthermore, the KL-G method achieves 13%–15% improvement over the upper confidence bound (UCB) method [36] and around 16%–18% over random sampling RS.
- Table 2 demonstrates the estimation error of the regression model parameters, averaged over different artificial datasets for all the considered active learning strategies. Our proposed balancing strategies achieving high utility (revenue) gain such as KL-G in both pool and synthesis settings, UoS-1 and UoS-2, are not the best performing methods in terms of the model estimation error. However, these methods eventually yield a better model estimation than the baselines including: greedy sampling and UCB as indicated in Table 2. Furthermore, the main target is utility (revenue) optimization and the model estimation is a necessary but secondary objective. Moreover, the other proposed balancing strategies such as KL-G, MI-G and MMI-G have comparable performance to the baselines.
- For the real datasets, it can be observed from Table 10 that our proposed first variant of the uncertainty of strategy (UoS-1) is the best performing method in terms of the revenue gain. Although G-Synth has the same average revenue gain as UoS-1 method’s gain, the UoS-1 method has lower estimation error rates than G-Synth. As mentioned in Section 5.4, the UoS-1 method accounts for the model uncertainty to control the exploration window (see Equation (72)). In addition to its promising performance, the UoS method is practically simple to implement.
- The two variants of our introduced balancing method, uncertainty of strategy (UoS), achieve significant performance in terms of the achieved revenue gain as indicated in Table 1 and Table 10 for synthetic and real datasets, respectively. The major reason for the significant performance of the UoS method is that it accounts for the uncertainty of the selection criterion itself. Furthermore, this method combines the exploration and exploitation probabilistically like the UCB and the PEE methods.
- Regarding the proposed PEE methods: KL-G, MI-G, MMMI-G and ME-G, they produce substantial performance in terms of the achieved revenue gain for synthetic and real datasets, as shown in Table 1 and Table 10, respectively. Specifically, the KL-G method is the best performing method in terms of the achieved revenue gain for the synthetic datasets (see Table 1).
- Furthermore, for the results of the real datasets presented in Table 10, the KL-G, MI-G, MMI-G and ME-G methods are of the top-10 strategies with respect to the achieved revenue gain. Moreover, for the model estimation error, they are comparable to the considered baselines. However, the KL-G variants provide competitive model estimation for both synthetic and real datasets as shown in Table 1 and Table 10, respectively.
- There are three major reasons for the promising results of the PEE strategies: KL-G, MI-G, MMI-G and ME-G. First, these strategies are based on information theoretic concepts: Kullback–Leibler divergence [44] and entropy [43], as described in Section 5.2. Second, these methods adopt a probabilistic approach for balancing the exploration and exploitation as presented in Section 5.4, unlike the UCB method. The third reason is that the employed exploration strategies perform an effective exploration since they take into account the information of the unlabeled samples and the model uncertainty.
- In addition to our proposed strategies of the probabilistic-based exploration-exploitation (PEE) method, we have extended two more PEE methods combining uncertainty sampling [1] and random sampling, to perform exploration, with greedy sampling for exploitation. We experiment these two baselines for comparison purposes. For synthetic datasets, it could be noticed from Table 2 that the RS-G method with both versions, pool-based and query synthesis, achieves comparative model estimation. However, the RS-G method compromises the achieved revenue gain as indicated from Table 1, since it obtains a revenue gain that is around 3%–4% below the top method, KL-G-Pool. Similarly, for the real datasets’ results presented in Table 10, the two methods US-G and RS-G obtain accurate model estimate, however both of these methods compromise the achieved revenue.
- These results essentially elucidate the significance of our proposed information-theoretic exploration strategies presented in Section 5.2. Although the same exploitation method is used, the greedy sampling and the same probabilistic approach, the PEE method, is followed for combining exploration and exploitation (see Section 5.4), the proposed methods, specifically, KL-G and MMMI-G, exhibit better performance than the US-G and RS-G methods in terms of the achieved revenue, which is the main target.
  Furthermore, our proposed methods obtain model estimation performance close to the US-G and RS-G methods, for both synthetic and real datasets as presented in Table 1 and Table 10, respectively. The other two proposed methods MI-G and ME-G, also outperform the RS-G and US-G methods, for real datasets and produce comparable performance for the synthetic datasets.
  The reason for the performance preeminence of our proposed information theory-based strategies over the US-G and RS-G methods in terms of revenue gain is that the proposed methods essentially exploit the potential information of the unlabeled data and the model uncertainty. Moreover, these strategies not only improve the model estimation error but also query representative data samples that minimize the model uncertainty, which promotes the exploitation performance.
- From Table 10, it can be noticed that the greedy sampling performs well in the real datasets, since the considered real datasets have very low noise, expressed in terms of $\hat{\sigma }$ in Table 7. Also, the UCB baseline [36] performs comparably well on the real datasets due to the datasets’ robustness. Similarly for artificial datasets, Table 1 shows that for the low noise datasets, having σ=5%, both of the greedy sampling and the UCB methods perform quite well, comparable to the best performing method, our proposed balancing method UoS-1. On the other hand, for the noisy datasets where σ=40%, both of the greedy sampling and the UCB methods result in poor performance, in terms of the gained revenue. This could be apparently observed from Table 1.
- Regarding the other developed pure exploitation method, namely the expected value of information (EVPI), it could be noticed from Table 2 and Table 10, the EVPI strategy results in better model estimation than the greedy sampling as EVPI chosen samples incur some diversity unlike the points chosen by the greedy method which essentially queries points maximizing the utility function. However, the greedy sampling, adequately, outperforms EVPI in case of low noise and for real datasets (see Table 1 and Table 10, respectively). On the other hand, for the noisy datasets, the EVPI approach, in both settings pool-based and query synthesis, attains larger revenue gain than the corresponding methods for greedy sampling as shown in Table 1 since the EVPI method is less myopic than the greedy sampling, so it is more immune to the noisy datasets.
- Concerning the random sampling (RS) baseline method, since it is a pure exploration strategy, convincingly, it does not achieve high revenue gains for synthetic and real datasets as indicated in Table 1 and Table 10, respectively. However, since random sampling could be regarded as a pure exploration methods, it, intuitively, performs well with respect to the model estimation error as shown in Table 1 and Table 10, for synthetic and real datasets, respectively. However, as previously mentioned, random sampling considerably jeopardizes the gained revenue.
- In this work, we perform a comparative empirical analysis between the pool-based and query synthesis active learning approaches. The empirical analysis considers both evaluation metrics, the achieved gained utility (revenue) and the percentage regression model error. As we mentioned in Section 7, we exclude the random sampling from this analysis since it is a passive learning method.
- Concerning the revenue gain, Table 3 and Table 13 demonstrate the average revenue for the strategies of each approach, for synthetic and real datasets, respectively. It could be observed that the query synthesis approach clearly outperforms the pool-based approach for both the artificial (with improvement around 3.5%) and real datasets (with improvement around 10%). The improvement is more significant in case of real datasets as will be discussed subsequently. Moreover, as indicated in Table 15, the query synthesis approach is more dominant within the top-10 strategies in terms of achieving revenue gain, for real datasets. Specifically, 76% of the top-10 strategies achieving revenue gain, belong to the query synthesis approach, whereby only 24% strategies are pool-based methods.
- Regarding the low noise synthetic data, both of pool-based and query synthesis approaches result in similar performance in terms of average revenue gain as shown in Table 3.
- On the other hand, in case of large noise level, it could be inferred from Table 3 that the query synthesis approach surpasses the pool-based one. In other words, the revenue gain improvement of the query synthesis over the pool-based approach is around 7.5%. In addition, the query synthesis methods have higher ranks than the pool-based ones in terms of the average revenue gain. Specifically, the ratio between the former and the latter is 55% to 45%, respectively. These results are persuasive since for noisy data, the pool of samples could be misleading, so querying a synthetic data sample in the global input space, that is not necessarily belonging to a specific set of data samples, would be more effective for optimizing a certain utility function.
- Regarding the model percentage error, the pool-based approach produces a slightly better model estimate for artificial datasets (see Table 4). However, the query synthesis methods have more advanced ranks than the pool-based methods as presented in Table 6, for both of the low and high noise levels.
- By examining the real datasets results, we can find that the query synthesis approach accomplishes less error rates than the pool-based approach as shown in Table 14. In addition, when investigating the top-10 strategies in terms of minimizing the model estimation errors in Table 16, one could observe that the query synthesis methods occupy 72% of the top-10 strategies, compared to 24% for the pool-based methods.
- The two real datasets, namely the sugar and coke datasets, described in Table 7 represent typical cases where the pool-based approach suffers from a major performance hurdle in terms of the obtained revenue gain as presented in Table 13. The reason for the poor performance of the pool based approach for these two datasets is that the available pool of data samples is limited and not representative enough. Furthermore, the available data samples do not contain the optimal price maximizing the target utility function, which is the gained revenue.
- For example, for the coke dataset, according to the linear demand model parameters presented in Table 7 and using the revenue equation (Equation (81)), the optimal price maximizing revenue is $\frac{-\hat{a}}{2\hat{b}}=74.24$. However, the mean and the standard deviation, of the available prices of this dataset are $\mu_{p}=22.96$ and $\sigma_{p}=3.2376$, respectively. Accordingly, the available prices of the pool are too far from optimal, that is why the pool-based strategies do not perform well on this dataset. The sugar dataset exhibits a very similar behavior as well. The lack of diversity in the pool is considered a serious drawback for the pool-based approach. In contrast, the query synthesis approach is not affected by such problem since the query synthesis approach chooses the data sample to be labeled from the entire input space and it is not restricted by the available pool of data (see Table 13 and Table 15).
- One could infer from Table 1 and Table 10 that the two variants of query synthesis approach with a predefined pool (see Algorithm 2) and without a predefined pool (see Algorithm 3), yield comparable performance for the different strategies. This is reasonable since both methods are logically equivalent, they only differ in implementation details. The query synthesis without a predefined pool approach, defined in Section 5.1 is essentially designed for the applications where it could be complicated to have a pool of representative data samples to be used for the information theory-based active learning strategies, namely KL, MI and MMI. In our experiments, we utilize the domain knowledge of the pricing data and construct a set of data samples belonging to the price range defined by the seller $\left[p_{min},p_{max}\right]$ to be used by the information theoretic methods: KL, MI and MMI.
- Finally, Table 11 and Table 12 show that increasing the initial training points enhances the performance of most of the active learning methods, in terms of both the revenue gain and the model estimation accuracy. These results are reasonable since having more initial data samples promotes the regression model’s accuracy, so the gained revenue is improved as well. In addition, Table 12 indicates that as the number of initial training samples increases, the performance of the different active learning methods gets closer to each other since the initial model estimate gets more robust. For the revenue gain, the query synthesis methods achieve similar performance as indicated in Table 11 for $N_{init}=10$. However, most of the pool-based methods do not achieve a significant performance improvement due to the limitation of the pool-based approach, previously discussed, for some real datasets, the sugar and coke datasets.

## 9. Conclusions

In this paper, we propose a novel active learning framework for optimizing a general utility function. Specifically, this work targets the class of problems incurring some trade-off between exploration and exploitation. We introduce several novel active learning methods for exploration, exploitation and for balancing both. The presented exploration strategies are essentially based on information theory concepts such as mutual information (MI), Kullback–Leibler divergence (KL) and model entropy (ME). Consequently, when combined with exploitation, such information theoretic exploration methods achieve promising performance in terms of the achieved utility and the learning model error as well. Furthermore, we develop new approaches for balancing exploration and exploitation such as the uncertainty of strategy (UoS) method that controls the exploration window according to the model uncertainty. In addition, we present another balancing method, utility minus entropy (UME) where the model entropy is explicitly modeled and augmented with the target utility function into one hybrid objective function to be optimized.

In this work, we investigate two main approaches of active learning, the pool-based approach which is widely used in active learning literature and the membership query synthesis approach. Moreover, we present an empirical analysis for comparing both approaches. The experiments show the exceptional performance of the query synthesis approach compared to the pool-based approach for the synthetic and real datasets. The compelling results for query synthesis approach could help boosting the active learning research towards employing the query synthesis approach.

We have applied the proposed framework to an operation research related application, namely, dynamic pricing with demand learning. However, our proposed framework can easily be adapted to other applications. We perform several experiments using synthetic and real datasets. In our experiments, we compare our proposed active learning strategies to several baselines and our presented strategies yield a significant performance improvement in terms of both aspects: the achieved gained revenue and the regression model error.

## Figures and Tables

**Table 1:** The average revenue gain of the active learning methods, over twelve synthetic datasets, using different noise levels σ. The strategies are sorted descendingly according to their average revenue gain over the two noise levels. The bold entries represent the maximum revenue gain per column (over all strategies).

Active Learning Strategy	σ=5%	σ=40%	Average over Noise Levels
KL-G-Pool	99.17%	**93.40%**	**96.28%**
KL-G-Synth-Nopool	98.17%	90.65%	94.41%
UoS-1-Synth	**99.64%**	88.71%	94.18%
RS-G-Pool	98.02%	89.78%	93.90%
MMI-G-Synth-Nopool	99.31%	88.41%	93.86%
MMI-G-Synth	99.17%	88.50%	93.84%
MI-G-Synth-Nopool	99.19%	88.34%	93.77%
UME-Synth	99.60%	87.11%	93.36%
UoS-2-Synth	99.17%	87.43%	93.30%
RS-G-Synth	95.38%	90.49%	92.94%
G-Synth	99.49%	85.25%	92.37%
KL-G-Synth	98.85%	85.30%	92.08%
ME-G-Synth	99.22%	83.48%	91.35%
MI-G-Synth	99.41%	82.97%	91.19%
MI-G-Pool	99.11%	83.18%	91.14%
MMI-G-Pool	98.93%	80.17%	89.55%
ME-G-Pool	99.17%	79.74%	89.46%
EVPI-Synth	89.12%	86.33%	87.73%
EVPI-Pool	85.97%	87.86%	86.92%
G-Pool	99.57%	68.28%	83.92%
UME-Pool	99.49%	68.13%	83.81%
US-G-Synth	85.15%	80.66%	82.91%
UCB-Pool	99.64%	64.19%	81.92%
UCB-Synth	99.48%	58.31%	78.90%
RS	77.61%	78.58%	78.09%
US-G-Pool	96.43%	57.67%	77.05%

**Table 2:** The average percentage model error of the active learning methods, over twelve synthetic datasets, using different noise levels σ. The strategies are sorted ascendingly according to their model estimation error over the two noise levels. The bold entries represent the minimum estimation error per column (over all strategies).

Active Learning Strategy	σ=5%	σ=40%	Average over Noise Levels
RS	0.67%	**8.74%**	**4.70%**
RS-G-Synth	1.49%	10.35%	5.92%
EVPI-Pool	3.10%	9.46%	6.28%
RS-G-Pool	2.75%	10.10%	6.42%
EVPI-Synth	3.47%	10.49%	6.98%
MI-G-Pool	3.84%	11.11%	7.48%
KL-G-Synth-Nopool	2.61%	13.56%	8.09%
KL-G-Pool	2.88%	14.73%	8.81%
US-G-Synth	**0.64%**	17.64%	9.14%
UCB-Synth	4.38%	14.48%	9.43%
UoS-1-Synth	5.38%	15.20%	10.29%
KL-G-Synth	2.05%	18.90%	10.47%
UoS-2-Synth	6.02%	15.77%	10.90%
UME-Pool	2.63%	19.70%	11.17%
US-G-Pool	5.02%	17.53%	11.27%
G-Pool	3.64%	19.31%	11.48%
ME-G-Pool	5.14%	18.08%	11.61%
MI-G-Synth	3.42%	23.01%	13.21%
MMI-G-Pool	5.02%	22.76%	13.89%
MMI-G-Synth-Nopool	3.27%	25.01%	14.14%
MMI-G-Synth	4.11%	27.14%	15.62%
MI-G-Synth-Nopool	4.21%	27.54%	15.87%
UME-Synth	5.28%	26.66%	15.97%
G-Synth	5.24%	27.82%	16.53%
ME-G-Synth	6.02%	27.37%	16.69%
UCB-Pool	4.64%	30.13%	17.39%

**Table 3:** The average revenue gain for the pool-based versus query synthesis approaches, over twelve synthetic datasets, using different noise levels σ. The bold entries represent the maximum average revenue gain per row (over the two active learning approaches).

Dataset	Noise Level σ	Pool-Based	Query Synthesis
a=100, b=−0.5	σ=5%	**98.66%**	98.36%
	σ=40%	80.44%	**80.85%**
a=100, b=−1	σ=5%	**96.41%**	95.24%
	σ=40%	88.92%	**90.86%**
a=100, b=−2	σ=5%	98.45%	**98.47%**
	σ=40%	83.80%	**91.81%**
a=1000, b=−0.5	σ=5%	97.71%	**97.99%**
	σ=40%	56.24%	**78.17%**
a=1000, b=−1	σ=5%	**97.49%**	96.25%
	σ=40%	**89.12%**	82.93%
a=1000, b=−2	σ=5%	96.59%	**97.84%**
	σ=40%	64.92%	**84.16%**
Average	σ=5%	**97.55%**	97.36%
Average	σ=40%	77.24%	**84.80%**
Average		87.40%	**91.08%**

**Table 4:** The average percentage model error for the pool-based versus query synthesis approaches, over twelve synthetic datasets, using different noise levels σ. The bold entries represent the minimum average estimation error per row (over the two active learning approaches).

Dataset	Noise Level σ	Pool-Based	Query Synthesis
a=100, b=−0.5	σ=5%	**3.21%**	4.60%
	σ=40%	**12.59%**	26.55%
a=100, b=−1	σ=5%	2.79%	**2.51%**
	σ=40%	**6.39%**	9.01%
a=100, b=−2	σ=5%	4.73%	**2.63%**
	σ=40%	**16.58%**	20.75%
a=1000, b=−0.5	σ=5%	4.03%	**2.68%**
	σ=40%	**14.33%**	17.72%
a=1000, b=−1	σ=5%	4.79%	**3.97%**
	σ=40%	31.24%	**28.39%**
a=1000, b=−2	σ=5%	**3.64%**	6.66%
	σ=40%	22.61%	**17.95%**
Average	σ=5%	3.87%	**3.84%**
Average	σ=40%	**17.29%**	20.06%
Average		**10.58%**	11.95%

**Table 5:** The percentage of strategies in the top-10 strategies achieving revenue gain belonging to the pool-based approach versus the query synthesis approach, over twelve synthetic datasets, using different noise levels σ. The bold entries represent the maximum percentage per row (over the two active learning approaches).

Dataset	Noise Level σ	Pool-Based	Query Synthesis
a=100, b=−0.5	σ=5%	**50.00%**	**50.00%**
	σ=40%	30.00%	**70.00%**
a=100, b=−1	σ=5%	**50.00%**	**50.00%**
	σ=40%	40.00%	**60.00%**
a=100, b=−2	σ=5%	**50.00%**	**50.00%**
	σ=40%	**60.00%**	40.00%
a=1000, b=−0.5	σ=5%	**50.00%**	**50.00%**
	σ=40%	**70.00%**	30.00%
a=1000, b=−1	σ=5%	**60.00%**	40.00%
	σ=40%	20.00%	**80.00%**
a=1000, b=−2	σ=5%	**50.00%**	**50.00%**
	σ=40%	**50.00%**	**50.00%**
Average	σ=5%	**51.67%**	48.33%
Average	σ=40%	45.00%	**55.00%**
Average		48.33%	**51.67%**

**Table 6:** The percentage of strategies in the top-10 strategies achieving the least regression model error, belonging to the pool-based approach versus the query synthesis approach, over twelve synthetic datasets, using different noise levels σ. The bold entries represent the maximum percentage per row (over the two active learning approaches).

Dataset	Noise Level σ	Pool-Based	Query Synthesis
a=100, b=−0.5	σ=5%	30.00%	**70.00%**
	σ=40%	20.00%	**80.00%**
a=100, b=−1	σ=5%	**50.00%**	**50.00%**
	σ=40%	40.00%	**60.00%**
a=100, b=−2	σ=5%	**60.00%**	40.00%
	σ=40%	40.00%	**60.00%**
a=1000, b=−0.5	σ=5%	**70.00%**	30.00%
	σ=40%	20.00%	**80.00%**
a=1000, b=−1	σ=5%	40.00%	**60.00%**
	σ=40%	40.00%	**60.00%**
a=1000, b=−2	σ=5%	20.00%	**80.00%**
	σ=40%	**60.00%**	40.00%
Average	σ=5%	45.00%	**55.00%**
Average	σ=40%	36.67%	**63.33%**
Average		40.83%	**59.17%**

**Table 7:** A description for the real-world datasets.

Dataset	Size	$\hat{a}$	$\hat{b}$	$\hat{\sigma }$	Mean of Prices $\mu_{p}$	Standard Deviation of Prices $\sigma_{p}$
Transport	41	41.3778	−0.1378	3.3902	145.00	90.8295
Beef	91	30.0515	−0.0465	0.5670	250.44	37.01
Sugar	18	1.3576	−0.3184	0.0292	1.005	0.0824
Spirits	69	4.4651	−1.2723	0.0573	2.1184	0.2089
Coke	20	50.5700	−0.3406	1.9319	22.96	3.2376

**Table 8:** The revenue gain for the different active learning strategies using the five real datasets. The bold entries represent the maximum revenue gain per column (over all strategies).

Active Learning Strategy	Transport	Beef	Sugar	Spirits	Coke
G-Pool	98.53%	99.61%	79.17%	99.23%	63.49%
G-Synth	98.85%	**99.81%**	99.25%	99.08%	98.59%
EVPI-Pool	84.65%	94.28%	72.31%	94.32%	51.90%
EVPI-Synth	**99.38%**	87.95%	89.02%	86.11%	97.50%
UCB-Pool	98.94%	99.60%	79.17%	99.26%	63.49%
UCB-Synth	98.76%	99.77%	98.72%	99.44%	98.41%
MI-G-Pool	98.52%	99.55%	79.01%	99.43%	63.08%
MI-G-Synth	98.62%	99.04%	98.58%	99.26%	97.14%
MI-G-Synth-Nopool	98.75%	99.50%	98.73%	99.23%	97.27%
MMI-G-Pool	98.19%	99.57%	79.06%	99.40%	63.01%
MMI-G-Synth	98.51%	99.43%	98.51%	99.29%	96.82%
MMI-G-Synth-Nopool	98.16%	99.40%	98.37%	99.39%	97.19%
KL-G-Pool	98.70%	99.22%	78.79%	99.32%	63.05%
KL-G-Synth	99.10%	98.42%	99.00%	98.77%	99.13%
KL-G-Synth-Nopool	98.19%	98.66%	**99.41%**	98.62%	99.21%
ME-G-Pool	98.83%	99.58%	79.03%	98.86%	63.09%
ME-G-Synth	98.88%	99.47%	98.59%	99.35%	97.33%
US-G-Pool	95.53%	99.74%	79.15%	98.18%	63.46%
US-G-Synth	84.86%	86.34%	89.95%	93.42%	86.53%
RS-G-Pool	97.84%	98.94%	78.57%	99.28%	62.81%
RS-G-Synth	95.40%	97.00%	97.17%	96.22%	96.15%
UoS-1-Synth	98.61%	99.69%	99.31%	99.27%	98.70%
UoS-2-Synth	98.01%	99.70%	98.72%	**99.50%**	99.15%
UME-Synth	98.63%	99.68%	93.60%	99.15%	**99.85%**
UME-Pool	98.53%	99.53%	78.90%	99.14%	62.38%
RS	78.54%	93.45%	71.89%	94.12%	52.10%

**Table 9:** The percentage error of regression model parameters for the different active learning strategies for the real datasets. The bold entries represent the minimum estimation error per column (over all strategies).

Active Learning Strategy	Transport	Beef	Sugar	Spirits	Coke
G-Pool	7.19%	5.36%	4.56%	3.36%	1.71%
G-Synth	9.60%	3.36%	1.02%	6.50%	1.31%
EVPI-Pool	2.24%	2.26%	3.62%	**0.43%**	2.34%
EVPI-Synth	4.62%	**0.52%**	1.17%	5.78%	1.53%
UCB-Pool	6.14%	4.89%	4.56%	3.28%	1.95%
UCB-Synth	9.15%	3.47%	1.45%	4.36%	1.44%
MI-G-Pool	7.18%	4.60%	3.66%	3.22%	1.79%
MI-G-Synth	9.76%	1.87%	1.16%	3.98%	1.89%
MI-G-Synth-Nopool	9.34%	3.47%	1.41%	5.17%	1.07%
MMI-G-Pool	10.78%	4.69%	4.26%	2.77%	1.65%
MMI-G-Synth	11.45%	2.35%	1.09%	4.90%	1.01%
MMI-G-Synth-Nopool	11.81%	4.07%	1.11%	3.79%	1.71%
KL-G-Pool	3.95%	4.87%	3.37%	3.42%	1.94%
KL-G-Synth	4.16%	1.59%	1.91%	0.70%	1.57%
KL-G-Synth-Nopool	8.50%	2.21%	1.60%	2.23%	1.54%
ME-G-Pool	5.64%	4.44%	3.81%	4.14%	1.70%
ME-G-Synth	8.84%	3.42%	0.94%	3.41%	1.81%
US-G-Pool	10.23%	2.87%	4.12%	1.48%	1.86%
US-G-Synth	3.17%	0.82%	**0.50%**	0.77%	**0.85%**
RS-G-Pool	6.23%	2.50%	5.38%	1.70%	3.41%
RS-G-Synth	2.22%	1.02%	1.00%	0.92%	1.10%
UoS-1-Synth	10.38%	4.10%	1.35%	3.04%	2.24%
UoS-2-Synth	10.86%	3.39%	1.50%	3.57%	1.05%
UME-Synth	9.93%	3.30%	0.59%	4.64%	1.05%
UME-Pool	8.16%	4.53%	7.35%	3.14%	1.94%
RS	**1.49%**	1.46%	2.59%	1.43%	2.72%

**Table 10:** The average results of active learning methods in terms of the average revenue gain and average percentage error, over the five considered real datasets. The strategies are sorted descendingly according to their average obtained revenue gain. The bold entries for the first column represent the maximum average revenue gain over all strategies and the bold entries for the second column represent the minimum estimation error over all strategies.

Active Learning Strategy	Average Revenue Gain	Average Model Percentage Error
UoS-1-Synth	**99.12%**	4.22%
G-Synth	**99.12%**	4.36%
UCB-Synth	99.02%	3.97%
UoS-2-Synth	99.02%	4.07%
KL-G-Synth	98.88%	1.99%
KL-G-Synth-Nopool	98.82%	3.22%
ME-G-Synth	98.72%	3.68%
MI-G-Synth-Nopool	98.70%	4.09%
MI-G-Synth	98.53%	3.73%
MMI-G-Synth	98.51%	4.16%
MMI-G-Synth-Nopool	98.50%	4.50%
UME-Synth	98.18%	3.90%
RS-G-Synth	96.39%	1.25%
EVPI-Synth	91.99%	2.72%
US-G-Synth	88.22%	**1.22%**
UCB-Pool	88.09%	4.16%
G-Pool	88.01%	4.44%
MI-G-Pool	87.92%	4.09%
ME-G-Pool	87.88%	3.95%
MMI-G-Pool	87.85%	4.83%
KL-G-Pool	87.82%	3.51%
UME-Pool	87.70%	5.02%
RS-G-Pool	87.49%	3.84%
US-G-Pool	87.21%	4.11%
EVPI-Pool	79.49%	2.18%
RS	78.02%	1.94%

**Table 11:** The average revenue gain of active learning methods versus different number of initial training points $N_{init}$, averaged over the five considered real datasets. The bold entries represent the maximum average revenue gain per row (over the different number of initial training points $N_{init}$).

Active Learning Strategy	$N_{\mathrm{init}}=3$	$N_{\mathrm{init}}=5$	$N_{\mathrm{init}}=10$
G-Pool	88.01%	88.14%	**88.14%**
G-Synth	99.12%	99.46%	**99.82%**
EVPI-Pool	79.49%	**80.87%**	80.06%
EVPI-Synth	91.99%	94.81%	**97.33%**
UCB-Pool	88.09%	**88.21%**	88.19%
UCB-Synth	99.02%	99.47%	**99.82%**
MI-G-Pool	**87.92%**	87.68%	87.92%
MI-G-Synth	98.53%	98.85%	**99.21%**
MI-G-Synth-Nopool	98.70%	98.92%	**98.96%**
MMI-G-Pool	87.85%	**87.93%**	87.88%
MMI-G-Synth	98.51%	98.87%	**99.14%**
MMI-G-Synth-Nopool	98.50%	98.65%	**99.14%**
KL-G-Pool	**87.82%**	87.72%	87.65%
KL-G-Synth	98.88%	**99.08%**	98.47%
KL-G-Synth-Nopool	98.82%	98.40%	**99.00%**
ME-G-Pool	87.88%	**88.11%**	87.98%
ME-G-Synth	98.72%	98.95%	**99.31%**
US-G-Pool	87.21%	86.74%	**87.52%**
US-G-Synth	88.22%	86.88%	**88.33%**
RS-G-Pool	87.49%	87.64%	**87.77%**
RS-G-Synth	96.39%	95.76%	**97.22%**
UoS-1-Synth	99.12%	99.39%	**99.76%**
UoS-2-Synth	99.02%	99.12%	**99.30%**
UME-Synth	98.18%	98.13%	**99.20%**
UME-Pool	87.70%	**87.79%**	87.74%
RS	78.02%	79.15%	**79.30%**
Average	92.66%	92.87%	**93.24%**

**Table 12:** The average model percentage error of active learning methods versus different number of initial training points $N_{init}$, averaged over the five considered real datasets. The bold entries represent the minimum average estimation error per row (over the different number of initial training points $N_{init}$).

Active Learning Strategy	$N_{\mathrm{init}}=3$	$N_{\mathrm{init}}=5$	$N_{\mathrm{init}}=10$
G-Pool	4.44%	3.02%	**1.72%**
G-Synth	4.36%	2.99%	**1.90%**
EVPI-Pool	2.18%	1.35%	**1.07%**
EVPI-Synth	2.72%	**1.94%**	2.07%
UCB-Pool	4.16%	2.93%	**1.83%**
UCB-Synth	3.97%	2.56%	**1.77%**
MI-G-Pool	4.09%	2.37%	**1.79%**
MI-G-Synth	3.73%	2.11%	**1.12%**
MI-G-Synth-Nopool	4.09%	1.98%	**1.00%**
MMI-G-Pool	4.83%	3.16%	**1.65%**
MMI-G-Synth	4.16%	2.77%	**0.67%**
MMI-G-Synth-Nopool	4.50%	2.67%	**2.12%**
KL-G-Pool	3.51%	**2.43%**	2.76%
KL-G-Synth	**1.99%**	3.17%	2.37%
KL-G-Synth-Nopool	3.22%	**2.13%**	2.18%
ME-G-Pool	3.95%	2.56%	**1.51%**
ME-G-Synth	3.68%	2.78%	**1.38%**
US-G-Pool	4.11%	3.16%	**1.70%**
US-G-Synth	1.22%	1.47%	**1.04%**
RS-G-Pool	3.84%	1.83%	**1.67%**
RS-G-Synth	1.25%	1.53%	**1.08%**
UoS-1-Synth	4.22%	3.10%	**1.80%**
UoS-2-Synth	4.07%	2.61%	**1.75%**
UME-Synth	3.90%	3.48%	**1.97%**
UME-Pool	5.02%	3.91%	**1.59%**
RS	1.94%	1.46%	**0.98%**
Average	3.58%	2.52%	**1.63%**

**Table 13:** The average revenue gain for the pool-based approach versus query synthesis approach, using the five real datasets. The bold entries represent the maximum average revenue gain per row (over the two active learning approaches).

Dataset	Pool-Based Approach	Query Synthesis Approach
Transport	96.83%	**97.51%**
Beef	**98.96%**	97.59%
Sugar	78.32%	**97.13%**
Spirits	**98.64%**	97.74%
Coke	61.98%	**97.26%**
Average	86.94%	**97.45%**

**Table 14:** The percentage model error for the pool-based versus query synthesis strategies, using five the real datasets. The bold entries represent the minimum average estimation error per row (over the two active learning approaches).

Dataset	Pool-Based Approach	Query Synthesis Approach
Transport	**6.77%**	8.25%
Beef	4.10%	**2.60%**
Sugar	4.47%	**1.19%**
Spirits	**2.69%**	3.58%
Coke	2.03%	**1.41%**
Average	4.01%	**3.41%**

**Table 15:** The percentage of pool-based versus query synthesis strategies, ranked within the top-10 strategies achieving the highest revenue gain, using the five real datasets. The bold entries represent the maximum percentage per row (over the two active learning approaches).

Dataset	Pool-Based Approach	Query Synthesis Approach
Transport	30.00%	**70.00%**
Beef	**50.00%**	**50.00%**
Sugar	0.00%	**100.00%**
Spirits	40.00%	**60.00%**
Coke	0.00%	**100.00%**
Average	24.00%	**76.00%**

**Table 16:** The percentage of pool-based versus query synthesis strategies ranked within the top-10 strategies with respect to achieving the least percentage model error, using the five real datasets. The bold entries represent the maximum percentage per row (over the two active learning approaches).

Dataset	Pool-Based Approach	Query Synthesis Approach
Transport	**60.00%**	40.00%
Beef	30.00%	**70.00%**
Sugar	0.00%	**100.00%**
Spirits	**50.00%**	**50.00%**
Coke	0.00%	**100.00%**
Average	28.00%	**72.00%**
